# Dynamic Microstructured Thermoresponsive Interfaces for Label-Free Cell Sorting Based on Nonspecific Interactions

**DOI:** 10.1021/acsami.5c08747

**Published:** 2025-08-20

**Authors:** Ronaldo Badenhorst, Sergei V. Makaev, Mikhail Parker, Rostyslav Marunych, Vladimir Reukov, Agnieszka Będzińska, Olexandr Korchynskyi, Ostap Kalyuzhnyi, Dmytro Yaremchuk, Jaroslav Ilnytskyi, Taras Patsahan, Sergiy Minko

**Affiliations:** † Nanostructured Materials Lab, 1355 University of Georgia, Athens, Georgia 30602, United States; ‡ Laboratory of Experimental Biology and Department of Biochemistry & General Chemistry, Medical Faculty, 49726 Rzeszów University, Warzywna 1a Str., Rzeszów 35-959, Poland; § Regenerative Bioscience Center, Department of Textiles, Merchandising and Interiors, Athens, Georgia 30602, United States; ∥ 124297 Yukhnovskii Institute for Condensed Matter Physics of the National Academy of Sciences of Ukraine, 1 Svientsitskii str., Lviv 79011, Ukraine; ⊥ Institute of Applied Mathematics and Fundamental Sciences, Lviv Polytechnic National University, 12 S. Bandera Str., Lviv 79013, Ukraine

**Keywords:** label-free cell sorting, reconfigurable microstructured surface, cell adhesion, phase separation in thin films, DPD simulations, Monte Carlo simulations

## Abstract

Label-free cell sorting methods and materials are developed in this work. The microstructured thermoresponsive surfaces made of poly­(glycidyl methacrylate) (PGMA) and poly­(*N*-isopropylacrylamide-*co*-glycidyl methacrylate) (PNIPAM-*co*-GMA) are prepared by phase separation on the submicron scale in thin films and then cross-linked and covalently grafted to the substrate. PGMA domains are used for cell adhesion, while the PNIPAM-co-GMA matrix pushes cells off the surface at a temperature below the lower critical solution temperature (LCST). The microstructure formation and swelling–shrinking caused by changes in temperature are studied experimentally and by using dissipative particle dynamics computer simulations. Experiments with RAW 264.7 murine macrophage-like cells, NIH3T3/GFP murine fibroblasts, and HaCaT human skin keratinocytes (unlabeled and GFP-positive strains) demonstrate successful cell sorting based on weak and nonspecific interactions with reconfigurable thermoresponsive microstructured surfaces. Efficient sorting with a separation factor of >50 is achieved if the push-off force is adjusted to a level between the adhesive forces of the separating cells. This experimental finding is supported by Monte Carlo simulations of cell adsorption and detachment on the microstructured surfaces. The experiments and simulations show that efficient cell sorting is possible for weak to moderate cell adhesion to the surfaces. However, the method is not successful for very weak or very strong adhesion. We demonstrate that cell adhesion to the microstructured surfaces can be adjusted by changes in the conditions of the phase separation at the stage of film formation and by varying the incubation time of the cells on the microstructured surfaces.

## Graphical abstract

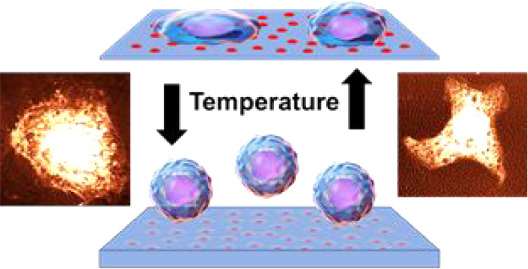

## 1 Introduction

Discrimination between cells and noncellular life forms (LF), such as viruses, in natural or artificial cell mixtures within their environment is crucial across various fields of biology, medicine, and biotechnology. Cell sorting methods are based on differences in the composition of cell membranes and the physical properties of cells. All cell sorting methods can be divided into two groups: label-based and label-free. Fluorescence-activated cell sorting^1^ (FACS) is commonly regarded as a major gold standard label-based method. This technology relies on the exclusive specificity of antibodies generated against different epitopes of cell surface markers. Unique fragments of polypeptides, carbohydrates, lipids, or other modifications within cell type-specific surface proteins serve as convenient antibody targets. Therefore, the isolation of specific cell types from complex multicomponent mixtures of different cells, such as circulating blood cellular components, requires expensive equipment and extensive sets of costly antibodies. An alternative antibody-based technique, magnetic-activated cell sorting (MACS), which uses magnetic beads conjugated with antibodies,^2^ does not require expensive equipment and is less time-consuming but is similarly dependent on costly antibodies. Unfortunately, all existing cell sorting procedures also generate a significant risk of mechanical cell damage and loss (around 7–14% for the MACS procedure and up to 70% in the case of the FACS process).^3^ In addition, many convenient surface protein targets represent signaling molecules. Highly specific protein–protein interactions of antibodies with such surface targets simultaneously generate a risk of activating the corresponding signaling pathways.

Alternative label-free methods explore differences in cell size,^4^ electrical charges,^5^ isoelectric points in an aqueous environment,^6^ and mechanical properties.^7^ Cell sorting based on cell density variations uses the density gradient centrifugation method.^8^ Among other label-free methods, microfluidic methods have gained significant interest.^9,10^ Microfluidic devices are classified into passive and active methods.^11^ Passive methods use flow and geometry for cell sorting, while active methods, such as magnetophoresis, acoustophoresis, and dielectrophoresis, refer to the cell’s response to applied external fields. However, quite often, the differences in the mentioned cell properties are not significant between different cells, and it is virtually impossible to use these approaches as universal label-free sorting methods.

Adhesion-based sorting methods have attracted special attention for cell sorting because of their potential scalability and simplicity, similar to chromatography, which is broadly applied for separating small molecules. Several approaches have been used and tested for cell affinity-based sorting. One of the approaches is to decorate the adsorbent surface or components of microfluidic devices with antibodies.^12,13^ In the latter case, cell sorting is based on differences in specific and nonspecific interactions. Generally, this approach is conceptually very similar to MACS label-based sorting. Because of the competition between specific and nonspecific interactions, the efficiency of cell sorting strongly depends on the cell mixture and overall cell concentrations. For highly asymmetric mixtures, where the cell of interest is a minority fraction, nonspecifically bound cells can occupy and block access to antibodies. Nonspecifically bound cells can be detached by liquid flow; however, its efficiency depends on the flow uniformity along the adsorbent surface and the surface concentration of the bound cells. Experimental studies have reported the efficiency of catching target cells ranging from 30%^12^ to 100%.^13^ The impact of nonspecific adsorption can be minimized by using microstructured surfaces (typically an array of micropillars).^14^ In the latter case, the contact surface area between the cell and the microstructured surface is minimal, making it easier to adjust the liquid flow threshold to remove nonspecifically bound cells. However, most of these methods involve noncontinuous and nonscalable separations, which imply limitations for many applications.

Using weaker adherent-specific motifs (moderately selective motifs) helped to realize a continuous cell sorting method.^15–18^ In one example, the target cells are periodically attracted in the flow by adhesive patterns, roll over the pattern, are released at the edge of the adhesive and nonadhesive patterns, and are attracted again by the next adhesive pattern so that the suspended waste is removed with the flow into a side channel while the target cells reside close to the microstructured surfaces and are extracted from the main channel. For example, a purity of 92% was achieved in 30 min for the separation of neutrophils from blood.^15^

Another less-explored approach to adhesion-based sorting relies on differences in nonspecific cell interactions.^19^ The essential advantage is that there is no need to use expensive antibodies or other types of selective ligands. However, this method is sensitive to cell-binding kinetics, which involves many biological aspects. Cell medium, extracellular matrix (ECM) proteins, and proteins secreted by cells can bind to the adsorbent surface and establish specific interactions via the integrin complex and focal adhesion formation.^20^ For example, such an approach was used to separate adherent and nonadherent cells^21^ while aiming to separate pseudonormal breast epithelial cells (MCF10A) from cancer cells (MCF7).^19^ It was found that the adhesion level plateaus for many cells after 1–2 h of incubation. For many applications, one round of cell sorting in such noncontinuous methods is insufficient for highly asymmetric cell mixtures. Multiple cycles of 1- to 2-h steps can take many days to reach a high separation level. A reasonable separation time was reported by Green and Murthy^22^ who described a nonspecific peptide-decorated flow setup that achieved 90% separation and removal of undesired cells, with a 45% loss of desired cells, in a 3-stage 1.5-h process while maintaining cell viability post separation.

The immensely successful developments of label-free methods provide various technical solutions for efficient cell sorting using microfluidic devices and micropatterned surfaces with highly and moderately selective motifs in periodic and continuous flow processes. An obvious advantage of the application of moderately selective motifs is the continuous technology. The drawbacks are the complex design of microfluidic devices and the limited possibilities for scale-up. The question of the potential of nonspecific adhesion-based label-free scalable sorting remains intriguing because of the limited literature.

There are at least two basic problems to be solved. In contrast to antibody-based cell sorting, sorting and separating affinity-based small molecules involve the adsorption–desorption equilibrium at the adsorbent surface. This method is efficient and inexpensive at different scales, ranging from laboratory analysis to industrial adsorption columns (column contact adsorbers). However, this method cannot be applied to cells because of the high contact surface area of the cell with the adsorbent. The latter is manifested in a very slow desorption process at the cell culture temperature.^23–25^ The adsorption energy of small molecules scales with a few *kT*, while the cell-adsorbent energy can approach hundreds to thousands of *kT* (where *kT* provides the energy scale of thermal fluctuations).^26^ The affinity-based adsorption equilibrium, based on thermal fluctuations, cannot be established for cells. An affinity-based sorting of cells could be achieved by overcoming the high desorption energy barrier using external energy sources, such as shear flow,^27^ ultrasound,^28,29^ or microfluidic devices discussed above. A precise and uniform adjustment of detachment forces using the shear forces of a liquid flux or ultrasound sources is difficult to achieve on large scales.

The second problem is related to cell biology, which is specifically important for adherent cells. Cell-surface interactions are kinetically subdivided into phases. The first phase is van der Waals forces, hydrogen bonds, hydrophobic interactions, and ionic forces,^30,31^ occurring within seconds. This is closely followed by the second phase, where integrin proteins associate with ligands in the cellular surroundings and bind to the surface exposed to cellular media containing proteins.^32,33^ Further eventual interactions involve phase three, characterized by cell flattening and surface spreading through cellular receptor clustering and cytoskeletal reconstruction. Finally, after 24 h, cells secrete ECM, proliferate, and form tissue.^34,35^ Adherent cells survive in suspension for only a short time. They grow on adhesive surfaces (typically amphiphilic). The most common method to harvest them is to use proteases to cut the protein complex’s connection to the surface. The “shaved” cells can then be transferred to another container with media, where they can synthesize membrane proteins and bind to the surface. The only short window for nonspecific interaction-based sorting of these cells is immediately after harvesting. In this case, biological processes do not likely interfere, and the cell can be considered a patchy elastic colloidal particle. However, the composition of the membrane molecules will depend on the harvesting method. Another biological aspect of concern is cell viability and turning on “wrong” signaling after sorting. Regarding this aspect, a weak adhesive interaction between cells and the substrate could be beneficial.

Recently, we reported one step in the direction of affinity-based cell sorting using microstructured surfaces composed of cell binding microdomains and thermoresponsive domains that undergo shrink-swell transitions at the lower critical solution temperature (LCST).^36^ Our concept refers to replacing the shear force of a liquid flux with an osmotic pushing-off force for cell detachment, assuming that this design is beneficial for uniform generation of cell detachment forces in high-volume cell sorting and manufacturing. The thermoresponsive domains were made of tethered poly­(*N*-isopropylacrylamide) (PNIPAM), which changes its interaction with the aqueous environment and undergoes phase transitions with temperature changes.^37^ The reversible phase transition in the thermoresponsive system was introduced by the change of the temperature around the LCST (32 °C for PNIPAM). This phase transition resulted in a reversible swelling or shrinking of the thermoresponsive domains. The latter process developed a push-off force to cause cell detachment. During the cold phase (30 °C), the cells were pushed off; during the warm phase (37 °C), the cells were adsorbed onto the surface. Affinity-based sorting was established when more strongly bound cells stayed on the surface. At the same time, weakly bound cells were pushed off the surface and resided in the solution. The binding domains contained RGD (arginyl-glycyl-aspartic acid) cell-adhesive motifs, which enabled the discrimination of cells with overexpressed integrin (cancer cells) from highly asymmetric mixtures with healthy cells. Importantly, the microstructured surface was designed appropriately to reach only the detachment of the cells that weakly interact with the surface and not to detach all cells that can be used for cell harvesting applications.^38^ In our previous work, we used RGD affinity motifs bound to the adhesive domains.^36^

The following step in the LF nonspecific sorting process was to model cell interaction with the microstructured dynamic surface using colloidal particles of a spherical or disk-like shape. We analyzed the effect of the geometry and dimensions of the microstructured domains to approach the LCST-induced swelling of PNIPAM domains, which was sufficient to weaken particle binding to the surface and push the particles off the surface in a controlled way.^39,40^

Here, we report on the next step for LF sorting by avoiding selective motifs to demonstrate the feasibility of sorting based on nonspecific cell adsorption on microstructured surfaces. In this case, the binding domains are made of cross-linked poly­(glycidyl methacrylate) (PGMA), and thermoresponsive domains are made of a cross-linked copolymer of PNIPAM and PGMA (PNIPAM-*co*-PGMA). PGMA is an amphiphilic polymer with a hydrophobic backbone and hydrophilic −OH side functional groups formed after opening the epoxy ring. We show that different types of mammalian cells can be sorted on this surface owing to differences in the interactions of the cells with PGMA domains. To verify the label-free sorting, we used specially labeled cells with recombinant proteins. The invention of jellyfish *Aequorea victoria* green fluorescent protein (GFP), along with its derivatives and mFruit series of similar proteins, generated a true revolution in live cell and tissue imaging.^41^ Different versions of the modified fluorescent proteins span the whole range of visible light. Such a wide spectrum allows us to identify and discriminate specifically labeled cells, proteins, or even tissues within a whole recombinant organism.^42^ The generation of recombinant proteins fused to parts of GFP allows the study of protein–protein interactions in live cells.^43^ Alternatively, the fluorescent protein-labeled cells can be sorted using conventional flow cytometric sorting without the involvement of expensive antibodies. Therefore, fluorescent proteins have become highly convenient for the visualization and quantification of reporter genes.

The ultimate goal of this work is to develop a scalable method for cell harvesting and sorting using cost-efficient cell sorting technologies and materials. In this work, we demonstrate that lithography for microstructured surfaces can be replaced with a more cost-efficient microphase separation method.

## 2 Experimental Part and Computer Modeling

### 2.1 Materials

Silicon wafers (Si-wafers) were purchased from University Wafer, Boston, MA, USA. (3-Glycidyloxypropyl)­trimethoxysilane (GOPTMS), *N*-isopropylacrylamide (NIPAM), glycidyl methacrylate (GMA), and azobis­(isobutyronitrile) (AIBN) were purchased from Millipore-Sigma, USA; 1,4-dioxane was purchased from Lab Alley, USA; toluene, methyl ethyl ketone (MEK), hexane, and ethanol were purchased from VWR Chemicals, USA; hydrogen peroxide (H_2_O_2_) and ammonium hydroxide (NH_4_OH) were purchased from Fischer Scientific, USA. Deionized (DI) water was prepared in the laboratory using ion exchange filters supplied by Evoqua, USA. Linear polyethylenimine (PEI), MW = 25 kg/mol was purchased from Polysciences Inc., Warrington, PA, USA. A lentiviral construct encoding enhanced GFP (eGFP) was kindly provided by Dr. Antoine A.F. de Vries, Leiden University Medical Center, Leiden, the Netherlands.

### 2.2 Synthesis of Polymers

PGMA and PNIPAM-co-GMA were synthesized in the laboratory as described below. PGMA homopolymer was synthesized using solution radical polymerization. GMA was dissolved in MEK (25 wt % monomer solution) and purified using an inhibitor removal column. AIBN was added to the solution at a 0.3 M concentration in the reaction mixture. The solution was purged with argon for 15 min; the polymerization was conducted for 2 h in a water bath at 40 °C and stopped by opening the cap, after which the polymer was precipitated 5 times from MEK in ethanol. The polymer yield was 50%; *M*_w_ = 570 kg/mol; *M*_w_/*M*_n_ = 3.0 (gel permeation chromatography data, GPC, dimethylformamide (DMF)).

A random copolymer PNIPAM-co-GMA was synthesized by using solution radical polymerization. Recrystallized from hexane and purified on an inhibitor removal aluminum oxide column, NIPAM and GMA, were dissolved in MEK at a ratio of NIPAM:GMA = 95:5 to prepare a 40 wt % monomer solution. AIBN was added to the solution at a 0.08 M concentration in the reaction mixture. The solution was purged with argon for 15 min; the polymerization was conducted for 2 h in a water bath at 40 °C and stopped by opening the cap, after which the copolymer was precipitated 5 times from MEK in hexane. The polymer yield was 80%. The molecular weight (*M*_w_) was 250 kg/mol and *M*_w_/*M*_n_ = 1.71 (GPC, DMF). The NIPAM:GMA ratio in the resulting copolymer was calculated by integrating the intensities of the amide groups from NIPAM and the ester groups from GMA on FTIR spectra (Figure S1) (PerkinElmer Frontier). For a 95:5 mol ratio of the feed solution, the calculated molar content of GMA was 5.3%.

Poly­(*N*-isopropylacrylamide) (PNIPAM) was synthesized in a manner similar to the PNIPAM-co-GMA copolymer, but without the addition of GMA. The polymer yield was 40%. *M*_w_ = 120 kg/mol *M*_w_/*M*_n_= 2.03 (GPC, DMF).

The phase behavior of PNIPAM-co-GMA aqueous solutions was tested by using turbidimetry (Figure S2). In contrast to PNIPAM homopolymer solutions with LCST= 32.5 °C, the PNIPAM-co-GMA copolymer (GMA 5.3% mol) has LCST = 28.5 °C.

The thermal characteristics of the bulk polymers (differential scanning calorimetry, DSC) reveal typical glass transition temperatures for cross-linked PGMA and PNIPAM at 60 and 135 °C, respectively (Figures S3, S4 and S5). The cross-linked PNIPAM-co-GMA copolymer and the homopolymer PNIPAM DSC plots are identical.

### 2.3 Cell Cultures and Media

Cell cultures: NIH3T3/GFP murine fibroblasts (hereafter referred to as 3T3) were generously donated by BioAesthetic, Durham, NC. These cells stably express the gene reporter for GFP for detection and identification. RAW 264.7 murine macrophage-like cells (hereafter referred to as RAW) were purchased from ATCC, USA. Human skin keratinocytes of the HaCaT cell line and HEK293T cells were kindly provided by Prof. Peter ten Dijke, Leiden University Medical Center, Leiden, the Netherlands. Dulbecco’s modified Eagle’s medium (DMEM) (Cat. No. D6429), fetal bovine serum (FBS) (Cat. No. ES-009-B), l-glutamine (Cat. No. TMS-002-C), sodium pyruvate (Cat. No. TMS-005-B), β-mercaptoethanol (Cat. No. ES-007-E), antibiotic-antimycotic (Cat. No. 15–240–112), trypsin-EDTA (Cat. No. T4049), and Dulbecco’s phosphate-buffered saline (DPBS) (Cat. No. D8537) were purchased from Millipore-Sigma, USA.

### 2.4 Amplification of Lentiviral Particles and Lentiviral Transduction of Cells

All the procedures for lentivirus particle amplification and infection were performed at the BSL2 laboratory facility. VSV-G pseudotyped lentiviral particles were amplified in HEK293T cells. Briefly, HEK293T cells at 60% confluency were transfected with a mixture of four plasmids: (1) Lentiviral construct; (2) VSV-G; (3) HIV-GAG/Pol; and (4) pRSV-Rev at a molar ratio of 2:1:1:1 using PEI. Forty-six hours after transfection, the supernatant from virus-producing cells was collected, cleared of cell debris by centrifugation at 6,000*g*, aliquoted, and frozen at 80 °C. A freshly thawed aliquot of lentiviral particles was added to model cells cultured in their complete growth medium supplemented with 10 μg/mL of DEAE dextran for 8 h. Forty-eight hours after transduction, the efficiently infected cells were selected with 1 μg/mL of puromycin for 3–5 days. After 5 days, the culture supernatant was collected for ELISA control specific for the p24 HIV envelope protein, and if negative, the cells were transferred to the BSL1 laboratory and used in the study.

### 2.5 Fabrication of Microstructured Thermoresponsive Coatings

*Functionalization of the surface of Si wafers*. After cutting Si wafers into 1 × 1 cm^2^ square samples, they were cleaned in piranha solution (1:1:1 ratio of ammonium hydroxide, DI water, and H_2_O_2_) for 60 min at 70 °C. The cleaned samples were rinsed with DI water and ethanol and dried under an argon flux. After washing and cleaning the Si wafers, they were immersed in a 1% GOPTMS solution in toluene for 10 h to functionalize the surface with epoxy groups for further use.

*Step 1. Fabrication of PGMA microdomains.* Solutions with three different ratios (1:5, 1:10, and 1:18) of 5% w/w PGMA and 4% w/w PNIPAM were prepared in dioxane. The solution was deposited on the GOPTMS-treated Si wafers using spin coating at 7000 rpm for 30 s. Following deposition, the sample was placed on a 150 °C heating plate for 3 min to ensure PGMA partial cross-linking (Figure S6).

*Step 2. Fabrication of the microstructured coatings*. The sample prepared in Step 1 was rinsed in DI water for 15 min to dissolve the PNIPAM matrix and dried under an argon flux. Two different 1% and 2% w/w PNIPAM-co-GMA solutions in ethanol were used to spin coat over the PGMA domain-decorated Si wafers at 7000 rpm. Following deposition, the samples were placed in a vacuum oven at 185 °C for 2 h to ensure cross-linking of PNIPAM-co-GMA and grafting it to the GOPTMS surface (Figure S7). The fabricated samples consisted of dual (PGMA and PNIPAM-co-GMA) domains and were fabricated using the ratios as follows: 1:5/1%, 1:5/2%, 1:10/1%, 1:10/2%, 1:18/1%, and 1:18/2%, which explain the ratios of PGMA and PNIPAM for the deposition of PGMA domains (in the numerator) and the concentration of PNIPAM-co-GMA for the deposition of PNIPAM-co-GMA domains (in the denominator). The surfaces used for later testing will be referred to as A1, A2, B1, B2, C1, and C2, with A corresponding to 1:5, B to 1:10, and C to 1:18 PGMA:PNIPAM solution ratios, while annotations 1 and 2 refer to PNIPAM-co-GMA solution concentrations. As a control, uniform single-component PGMA and PNIPAM-co-GMA films were fabricated.

*Plasma etching of microstructured dual domains.* The excess PNIPAM-co-GMA over PGMA domains was removed using vacuum plasma etching for 1 min with Harrick Plasma PDC-001 at maximum power (∼30 W, 0.8 mmHg air) (Figure S8). The etched PNIPAM-co-GMA thickness was determined from the analysis of SPM images taken before and after PNIPAM-co-GMA deposition. The etching time was adjusted based on the etching kinetics (Figure S9).

### 2.6 Characterization of the Microstructured Surface: Simulations

Understanding the details of the structure of the microstructured surfaces obtained in Step 1 was targeted using the dissipative particle dynamics (DPD) method,^44^ which allows for reaching the mesoscale while retaining principal chemical features on a coarse-grained level. The repeating units of PGMA and PNIPAM chains are treated as single soft beads of roughly 10 atoms. Explicit water is modeled as a set of separate beads. Such model chains were given the possibility to both be phase-separate and be grafted to the substrate. Details of the parameterization of the model can be found in the Supporting Information.

Swelling of the PNIPAM-co-GMA matrix was simulated while considering grafting to the cross-linked network on the substrate (GOPTMS-treated basal surface of the Si wafer in the experiments) and to PGMA domains that carry unreacted yet epoxy-functional groups. Computer simulations using the DPD method were used to analyze the potential effect of additional pinning of the matrix by PGMA domains. Details of the model can be found in the Supporting Information.

### 2.7 Characterization of the Microstructured Surfaces: Experiments

The samples of the microstructured surfaces were analyzed using scanning probe microscopy (SPM) with Dimension Icon and MultiMode 8 (Bruker) microscopes. The sample characterization was performed for the dry samples in air and in water at room temperature and at 40 °C. The scanning conditions in air were as follows: 10 × 10 to 40 × 40 μm scans with resolutions of 256 × 256 and 1024 × 1024 pixels in PeakForce Air mode using a TESP probe (spring constant ∼ 40 N/m); in water: 10 × 10 to 20 × 20 μm scans with resolutions of 256 × 256 and 512 × 512 pixels in PeakForce Fluid mode using a PNP-TR-Au probe (spring constant ∼0.08 N/m). The same sample was scanned at least four times: (1) after spin coating, short-time annealing, and washing out PNIPAM, assuming that short-time annealing (150 °C, 3 min) does not affect the structure of the PNIPAM domains, these SPM scans are considered to reflect the structure of PGMA domains formed during the phase separation stage; (2) after deposition of PNIPAM-*co*-GMA and long-time annealing (185 °C, 2 h), assuming that the structure initially changes and then cross-links; (3) after plasma treatment; and (4) in water above and below LCST. Not all samples were scanned in water at *T* > LCST, because no substantial differences were observed between samples scanned in air and water at *T* > LCST.

### 2.8 Cell Sorting Experiments

*Establishing the strength of cell attachment after 20 min of (short) incubation (cells used in the experiments: RAW, 3T3, HaCaT).* Approximately 20000 cells per 100 μL droplet of 37 °C DMEM were deposited on microstructure-coated Si-wafers preheated to 37 °C and glued to the bottom of a Petri dish. The droplet was placed directly on top of the wafer. Following deposition, the samples were incubated in a CO_2_ incubator at 37 °C for 20 min to let all cells to settle on the surfaces. Then, the wafer was immediately placed under an optical microscope (Olympus BX-51 microscope equipped with Tucsen TCC-3.3ICE-N camera under 5× magnification). Images were recorded under bright-field illumination and green fluorescent illumination. After collecting images of the attached cells at several locations (up to 7 for each wafer), the samples were washed with 2 mL of ice-cold PBS by pipetting and imaged under the microscope to determine the degree of cell detachment. The experiment was repeated with a flow-through system (Figure S10) using a peristaltic pump instead of pipetting, where the strength of the flow shear force could be estimated. PGMA-only and PNIPAM-only surfaces were used as controls. NOTE: since seeding timing was of high importance, cells were seeded not simultaneously but one wafer at a time.

*Establishing the strength of cell attachment after 16 h (long) incubation for the RAW, 3T3, and HaCaT cells.* For the overnight attachment study, cells were seeded on microdomain-coated Si wafers. The wafers were glued in a well of a 6-well plate. Aapproximately 40000 cells in 4 mL of 37 °C DMEM were seeded. After that, the plates were incubated overnight (approximately 16 h) in the CO_2_ incubator at 37 °C. The next day, Si wafers were imaged under the microscope, washed with ice-cold PBS by pipetting and using the flow-through system, and imaged again to determine the degree of cell detachment. NOTE: since the change in temperature might have affected cell detachment, wafers were investigated individually rather than simultaneously.

*Mixed cell sorting after 20-min (short) incubation.* The short incubation time protocol was repeated for mixtures of 3T3/RAW, 3T3/HaCaT, and HaCaT/RAW cells, which had about 20000 cells of each cell type (40000 total).

*Mixed cell sorting after 1-h (median) incubation.* Since HaCaT cells require a longer time to properly attach to PGMA surfaces, the sorting experiment was repeated for the 3T3/HaCaT and HaCaT/RAW cell mixture with 1-h incubation time.

*Mixed cell sorting after 16 h (long) incubation.* The long incubation time protocol was repeated for a mixture of 3T3/RAW, 3T3/HaCaT, and HaCaT/RAW cells. For the initial 3T3/RAW mixed seeding, we used 20000 3T3 and 10000 RAW cells (30000 total). For HaCaT/RAW, 20000 HaCaT and 10000 RAW cells were also used (30000 total). And for 3T3/HaCaT, 15000 cells of each were used in the experiment. This ratio was selected taking into account that the average doubling time of RAW cells is about 15 h, while 3T3 and HaCaTare 18–26 h and 26–28 h, respectively.

*Image analysis.* Images of the seeded cell cultures were analyzed by counting cells on the microstructured and control surfaces using ImageJ software, typically by thresholding the pictures after applying several filters (background subtraction, blurring, and segmentation). Bright-field pictures were used to count the total number of cells. GFP-modified cells fluoresce under blue light, emitting green light; therefore, green fluorescent pictures were used to count only the GFP-modified cells. The count of nonfluorescent cells was obtained by subtracting the GFP-modified cell counts from the total number of cells.

### 2.9 Monte Carlo Simulations of Cell Sorting

Adhesive domains of the microstructured surface were modeled as an array of cylindrical objects aligned in a plane along the surface, with their axes oriented orthogonally to it. All domains are assumed to have equal dimensions (both height and diameter) and are randomly distributed over the surface without overlap. Positions and orientations of the domains are fixed throughout the simulation. The top domain surfaces exhibit adhesive interactions with the cell surface, and the adhesion energy depends linearly on the contact area between the cell and domain surfaces. The space between domains along the surface is filled with a polymer phase. In the collapsed state, the polymers do not interact with the cell. In the swollen state, the polymer phase rises above the domain level and repels the cell from the surface. The cell is modeled as spherical particles while suspended in solution but may also deform upon contact with the surface, increasing its contact area at the cell-domain interface. The contact period is assumed to be short enough that the cell’s overall shape and dimensions remain unchanged. The local deformation at the interface between the cell and domains is represented as a flat circular intersection of the cell sphere, with the diameter of this section determined by the distance between the cell center and the cell-domain interface. The extent of deformation is limited by the minimum available distance between the cell center and the cell-domain interface. Since a cell can simultaneously contact multiple domains, the attractive interaction between the cell and the surface results from the sum of interactions between the cell and all domains in contact. The cell can immerse into the swollen polymer phase until it is stopped by contact with a domain surface and reaches its maximum extent of deformation. The energy of the repulsive interaction is determined by the circular intersection between the cell sphere and the polymer phase surface, which is located above the domain surface. The intersection between the cell sphere and the polymer phase should exclude regions occupied by the domains, regardless of how much higher the polymer phase surface is compared with the domain surfaces. The cells interact with each other as hard spheres. In addition to the attractive and repulsive interactions, there is also a hard-wall interaction between the cells and surface domains. Since all gaps between the domains are smaller than the cell size, the cell cannot penetrate below the level of the domain’s top surface. Due to being denser than the surrounding solution, the cells precipitate onto the patterned surface under gravity. The model incorporates gravity as an external field that drives the cells toward the microstructured surface, with the associated interaction energy varying linearly with the distance between the cell centers and the surface. Cells exhibit random motion resulting from disturbances induced by intrinsic sources (e.g., fluid flow, shear gradients, hydrodynamic instabilities, and substrate vibration). These sources are nonthermal, but in terms of our simulation, they play the same role as fluctuation noise produced by the temperature. At the same time, real thermal fluctuations are considered negligibly small for such large objects as cells. Details of the model and methods are discussed in the Suppporting Information.

## 3 Results and Discussion

### 3.1 General Concept

The concept of cell sorting using microstructured thermoresponsive surfaces or coatings is illustrated in Figure 1. The coating consists of two different microdomains. One domain type (made of PGMA) is adhesive to cells (nonspecific weak adhesion), while the second domain type is a matrix made of PNIPAM-*co*-GMA, which pushes off the cells from the surface upon swelling at a temperature below the LCST. Importantly, efficient cell detachment can be approached if the PNIPAM-co-GMA domains swell at least 25 nm above the level of PGMA domains, as discussed elsewhere.^39^ This characteristic length is defined by the size of the cell integrin complex responsible for cell binding and can be applied to cells with the integrin complex intact. However, when the integrin complex has not yet been regenerated for recently harvested cells, this characteristic length may change.

**Figure 1:**
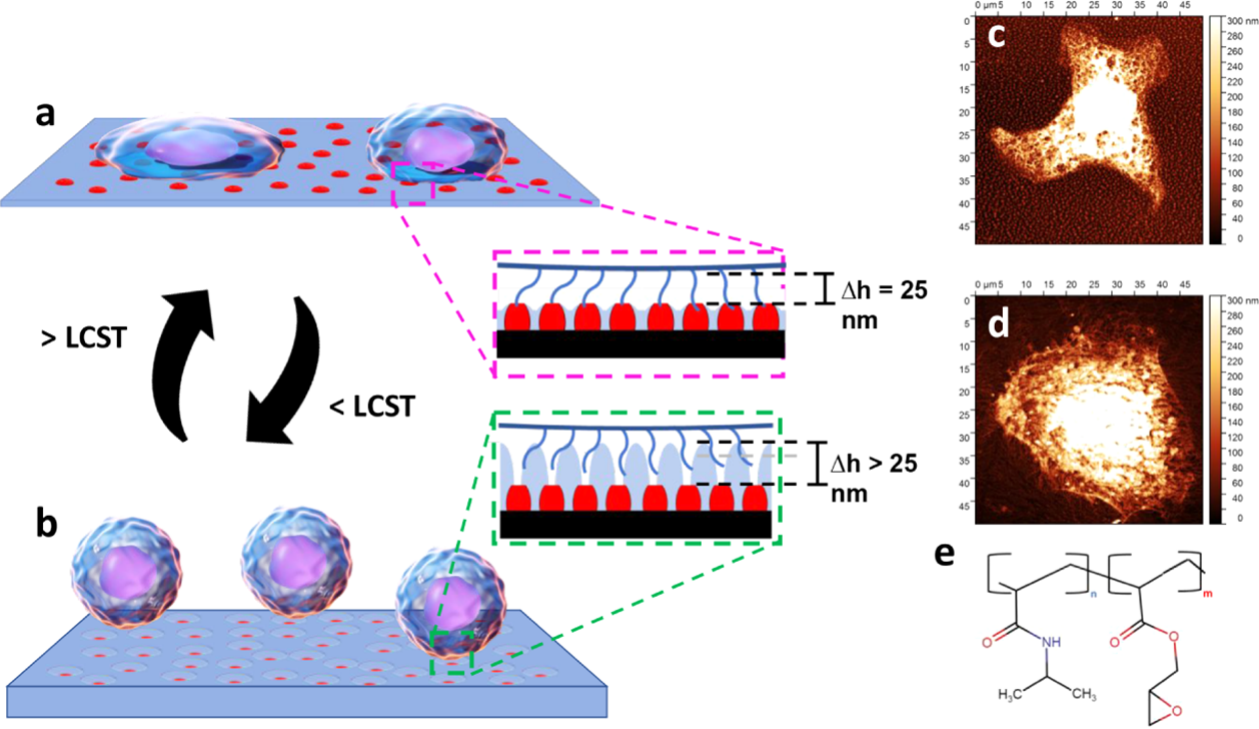
Concept of cell sorting. (a,b) Schematic of the microstructured surface, with red indicating PGMA domains and blue representing the PNIPAM-co-GMA matrix as it undergoes changes with temperature and achieves varying detachment of different cell types to promote cell sorting. (a) The PNIPAM matrix is in the collapsed state and facilitates nonspecific cell adhesion to PGMA domains at regular incubation temperatures above LCST. (b) The PNIPAM matrix swells to push off weaker adhered cells when the temperature drops below LCST. The insets (a) and (b) underline the critical length scale characteristics for swelling of the PNIPAM domains: 25 nm is the length of the cell integrin binding complex. (c,d) The insets are SPM images of the cells (c) bound to the microstructured surface and (d) bound to the reference PGMA surface. (e) Chemical structure of the matrix copolymer with 5 wt % of GMA monomeric units.

Cell sorting based on their adhesiveness to the coating can be approached experimentally if weakly adhesive cell A is pushed off the surface by the swollen PNIPAM-co-GMA domains, while the stronger-bound cell B remains on the surface. Consequently, for cell sorting of a mixture of cells A and B, the push-off force (POF) generated by the swollen domains should be above the adhesive force of cell A (AdA) and below the adhesive force of cell B (AdB) or AdA < POF < AdB. This requirement is applied to the one-step or periodic sorting method, which includes the assumption that all cells are uniformly adhered to the surface. For a continuous process, the condition is POF > AdB > AdA. Obviously, this condition assumes the detachment of all the surface-bound cells but differs in the kinetics of the detachment. Additional requirements should be added to realize continuous sorting. However, this paper focuses on one-step sorting to enable it to serve as a precursor and optimization step for a future continuous process. Notably, the advantage of the thermoresponsive surface is the uniformity of the osmotic swelling, independent of the arrangement and geometry of the supporting basal surface.

### 3.2 Fabrication of the Microstructured Surfaces: Experiments and Simulations

Approaching the AdA < POF < AdB condition depends on the height ratio of the PNIPAM-co-GMA domain to the PGMA domains, the cross-linking density of PNIPAM-co-GMA domains, and the lateral dimensions of the domains or surface coverage by the domains. The first goal is to explore these three adjustable factors for the fabrication of microstructured coatings with tunable POF. The second goal is to replace costly lithographic methods with a simple and scalable method of fabrication based on the phase separation of two polymers during film formation, which can be applied to larger surface areas using spin coating or dip coating technologies. The microstructured surfaces were fabricated in two steps. In Step 1 (Figure S6), the mixture of PNIPAM and PGMA in dioxane was spin coated onto the surface of the Si wafer. The phase separation upon solvent evaporation results in the formation of microstructured films when the phase separation is frozen upon the vitrification of the polymers. The structure of the film and the domain dimensions depend on the miscibility characteristics of the polymers, their ratio in the mixture, the solvent, and the conditions of spin coating. Many of these parameters are found empirically in experiments. After deposition, only the PGMA domains were thermally partially cross-linked (to provide the PGMA domain stability in the following steps) with a short annealing time (3 min) at 150 °C, while PNIPAM was not cross-linked, purposely to allow subsequent steps. The ratio between the two polymers in solution was PNIPAM:PGMA > 1 to ensure the formation of a PNIPAM matrix and PGMA island domains.

An equivalent model system was obtained in the form of phase-separated, surface-tethered polymer chains under conditions of strong repulsion between the PGMA and PNIPAM polymers in the θ-solvent for a range of mass fractions, *f,* of PGMA. The result is illustrated with a sequence of snapshots at the substrate level in Figure 2. The PGMA and PNIPAM domains are displayed in orange and magenta, respectively. The solvent is not shown to avoid clogging.

**Figure 2:**
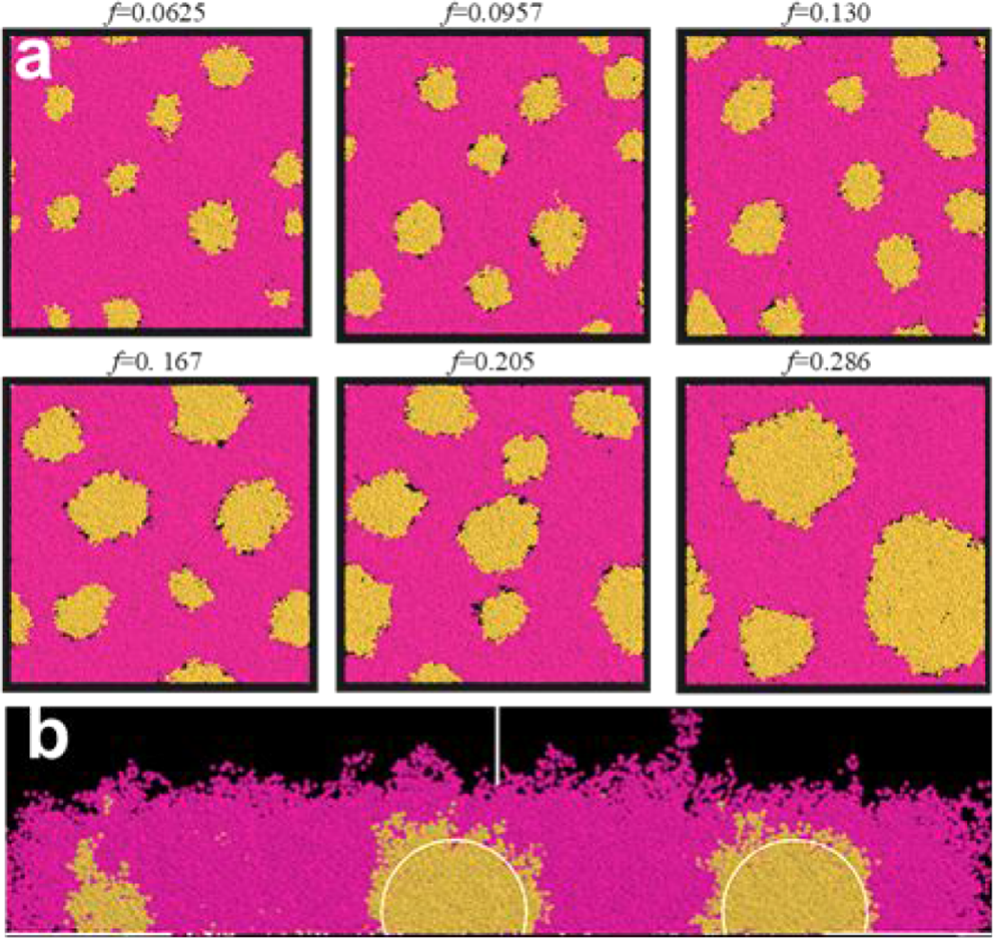
Computer simulation snapshots of the phase-separated PGMA/PNIPAM film in the form of a tethered polymer layer for different mass ratios of PGMA, *f*. Simulation time: 5 × 10^5^ DPD steps: (a) substrate-level view and (b) side view, *f* = 0.167. White circles represent the spherical cap shape of the PGMA domains.

In Step 2 (Figure S7), the phase-separated film was rinsed in water to extract PNIPAM, resulting in PGMA spherical cap structures decorating the surface. Then, different concentrations of PNIPAM-co-GMA solutions were used to refill the gaps between the PGMA domains using the spin coating method. Higher concentrations of the copolymer resulted in a thicker PNIPAM-co-GMA matrix. We experimentally found a range of concentrations to fabricate thin film coatings with different ratios of PGMA domain heights and PNIPAM-co-GMA matrix thicknesses. The deposition of PNIPAM-co-GMA resulted in the formation of a thin film coating over the PGMA domains, which blocked direct PGMA access. This thin layer was etched with plasma for 1 min (Figures S8 and S9) to remove it. We prepared a series of samples by varying the concentration of PNIPAM/PGMA mixtures in Step 1 to vary the size of the PGMA domains and their height. We varied the concentration of PNIPAM-co-GMA in Step 2 to vary the PGMA/PNIPAM-co-GMA height ratio (Table S1).

### 3.3 Characterization of Microstructured Surfaces

The fabricated microstructured surfaces were characterized using SPM after Step 1 (Figure S12) and after Step 2 in air and water at temperatures below and above the LCST. The representative SPM images and the corresponding cross-sections are shown in Figure 3. Notably, the swelling of PNIPAM-co-GMA in water at *T* > LCST is only 10–20%. Consequently, the images underwater at *T* > LCST and in the air are very similar. The bumpy surface of the coating at *T* > LCST with PGMA bumps (Figure 3a), seen in profile (Figure 3c), is transformed into a crater-like surface after the swelling of the PNIPAM-co-GMA matrix (Figure 3b), with the degree of swelling resulting in notable coverage of the PNIPAM domains (Figure 3c).

**Figure 3:**
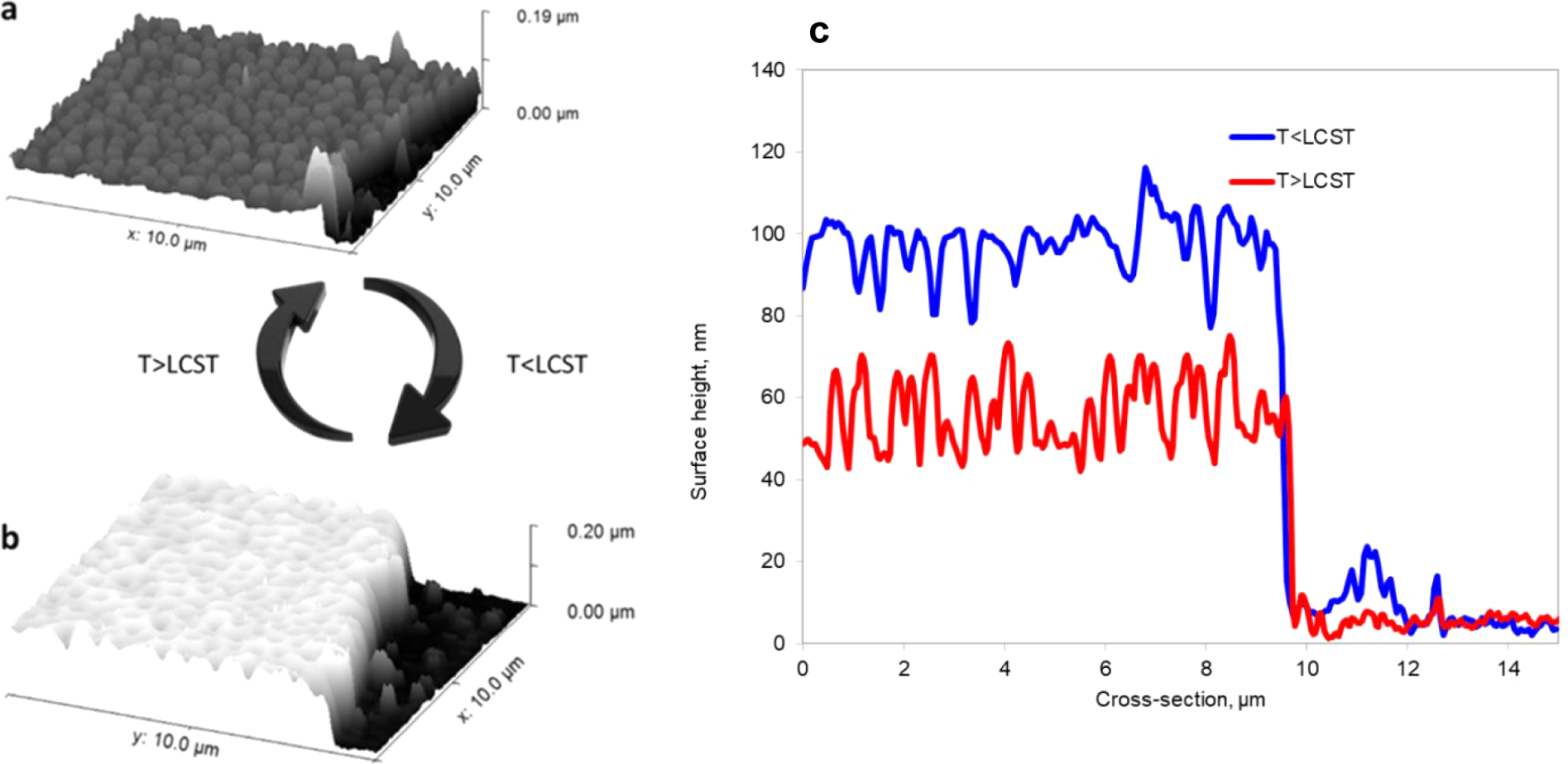
SPM image of a typical microstructured coating in water: (a) *T* > LCST and (b) *T* < LCST, and (c) the corresponding cross-sectional profiles.

The SPM data were analyzed to extract the dimensional characteristics of the microstructured surfaces. It was essential to obtain a statistical analysis of the major dimensional characteristics because of their critical role in cell sorting. In this analysis, we modeled the PGMA domains using the spherical cap geometry, schematically shown in Figure 4. The spherical cap model was used to characterize the microstructured surfaces after Step 1, which includes partial cross-linking, and after Step 2, which includes plasma treatment, to monitor different stages of the fabrication.

**Figure 4:**
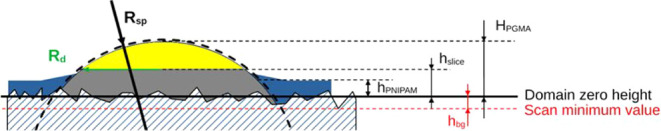
Schematic of the cross-section of a PGMA domain: h_bg_ is the roughness of the basal surface, typically 3–7 nm; h_slice_ and H_PGMA_ were measured from the scan minimum value, and then, h_bg_ was subtracted to set the background to zero. h_PNIPAM_ is the height of the PNIPAM-co-GMA matrix; h_slice_ is the height of a virtual slice, with the yellow-colored area above the slice; H_PGMA_ is the maximum height of the domain, taken as a median of maximum heights for all domains; R_sp_ is the sphere’s radius with the same curvature as the domain, taken as an average of the median largest and the median smallest curvature radii for all domains. R_d_ is the radius of a disc with the same projected area, estimated as a median for all domains.

The choice of spherical cap geometry was supported by the analysis of the PGMA domain shape using experimental and simulation data. The SPM images and simulation data were used to slice the PGMA domains parallel to the substrate plane and compare the experimental and simulation geometry with the geometry of the spherical cap (Table S1). We may draw several conclusions from this data. First, for the experimental data, the spherical cap approximation for the PGMA domains works very well as the individual radii, *R*_*sp*_, are very close for various slices of the same sample. The values of *R*_*d*_, measured in the experiment, and the values of *R*_*d*_ *’* for the spherical cap geometry are also very close. Second, the domains’ average radius and average height increase approximately linearly with the fraction of PGMA, *f*. The accuracy of the simulation data is lower, and we attribute this to the moderate system size. From the comparison of the experimental and simulation data (H_PGMA_), we found the length scaling factor of σ *≈* 9 nm (see the Supporting Information Modeling Section). Then, we note that matching the *R*_*d*_ values requires a factor about 4 times larger than that, namely, σ’ ≈ 36 nm. This means that the simulation domains are less “immersed” in the substrate than in the experimental structures, which are also visualized in Figure 2b. We can speculate that the latter is attributed to the early stage of phase separation in the case of simulations. Another possible reason for the discrepancy between the experimental and simulation results for the PGMA shape is the role played by the PGMA-substrate interaction. This can be addressed in future studies.

The several fabrication steps, including thermal annealing and plasma treatment, led to changes in the dimensions of the initially formed domains (Tables 1 and S2). The SPM scanning was repeated for the microstructured surfaces after plasma treatment to obtain the characteristics of the surfaces used for cell sorting. The dimensions of the domains in the microstructured surface fabricated in Step 2 are shown in Table 1. The height distributions for PGMA domains after Step 1 and Step 2 are shown in Figures S13 and S15, respectively. The height distribution of the PNIPAM domains is shown in Figure S14. The changes in the height distribution for the microstructured surfaces, caused by swelling of the PNIPAM-co-GMA matrix, are shown in Figure S16. The results show a very broad height distribution for PGMA domains and a narrow height variation for the PNIPAM-co-GMA matrix.

**Table 1:** Structural Characteristics of the Microstructured Surfaces^a^

Sample	H_PGMA_ air, nm	h_PNIPAM_, air, nm	h_PNIPAM_ below LCST, nm	Swelling ratio	d, nm	ρ, μm^–2^	A air, μm^2^	ρ×A
A0	104.5 (66.6)	-	-	-	494 (84)	3.9	-	-
A1	57.1 (13.6)	16.7 (2.9)	57.7 (6.8)	3.5	502 (96)	3.7	0.17 (0.10)	0.58
A2	72.8 (6.7)	51.5 (2.3)	113.2 (6.6)	2.2	532 (87)	3.5	0.125 (0.11)	0.51
B0	76 (19.7)	-	-	-	384 (65)	6.4	-	-
B1	41.5 (7.4)	19.4 (1.8)	40.0 (3.2)	2.1	377 (75)	6.5	0.076 (0.055)	0.49
B2	63.7 (7.0)	41.7 (1.8)	91.7 (5.9)	2.2	400 (68)	6.1	0.075 (0.055)	0.45
C0	52 (18.7)	-	-	-	357 (73)	7.0	-	-
C1	32.6 (5.8)	15.9 (1.5)	33.1 (4.2)	2.1	354 (72)	6.8	0.045 (0.033)	0.31
C2	58.6 (4.1)	45.1 (1.4)	95.3 (5.3)	2.1	374 (66)	7.2	0.055 (0.032)	0.40

a H_PGMA_ air is the median of the highest points of PGMA domain in air, with the interquartile range in parentheses; h_PNIPAM_ air is the height of PNIPAM layer in air, with the variance in parentheses; h_PNIPAM_ below LCST is the height of PNIPAM layer in H_2_O at 24 °C, with the variance in parentheses; d is the distance between domains centers, averaged over four nearest domains; ρ is the number of domains per 1 μm^‑2^; A is the median of the domain area above PNIPAM layer in air, with the interquartile range in parentheses; ρ × A is the surface coverage by PGMA domains exposed above PNIPAM at T>LCST. A, B, and C denote PGMA:PNIPAM-co-GMA ratio; 1 and 2 correspond to two different PNIPAM-co-GMA film thicknesses (Step 2); 0 denotes the samples received after phase separation, short annealing, and washing out PNIPAM (Step1).

### 3.4 Swelling PNIPAM Matrix

DPD simulations were performed to address two questions: (1) the effect of cross-linking on the swelling of the surface-grafted PNIPAM-co-GMA matrix and (2) the effect of the microstructured surface geometry and pinning (grafting to PGMA domains) on the swelling of the PNIPAM-co-GMA matrix.

The repeating units of PGMA and PNIPAM chains are treated as a single soft bead, each consisting of roughly 10 atoms, as shown in Figure S17. Details of the models are discussed in Figures S18–S20. Initially, we performed cross-linking of the PGMA domains. To this end, all its beads are considered initially active and accessible for cross-linking. It occurs with a probability of 0.1 if a pair of active beads touch or interpenetrate each other’s soft core. Once a cross-link is registered, both beads are exempt from subsequent cross-linking attempts. The cross-linking lasted 50 × 10^3^ DPD steps, and the number of cross-links saturated at the end. Because of the vast number of active GMA groups in PGMA, the PGMA domains are practically solidified. In the next step, the voids between PGMA domains are filled by the PNIPAM-co-GMA copolymer, which is also cross-linked. For this purpose, we assume 5% of its beads are of the GMA type.

To examine the effect of PNIPAM-co-GMA cross-linking on its thermoresponsive properties, we performed a simulation for the surface-grafted PNIPAM-co-GMA cross-linked in the collapsed state. Three grafting densities, ρ_*g*_ = 0.2, 0.4, and 0.6, were examined. The cross-linking fraction of PNIPAM-co-GMA was defined as ν_*cr*_ = 2*N*_*b*_/*N*_*pnipam*_100%, where *N*_*b*_ is the number of formed cross-linking bonds and *N*_*pnipam*_ is the total number of PNIPAM-co-GMA polymer beads. Here, the initial fraction of GMA was 30%, and uncross-linked beads transformed into PNIPAM after the needed ν_*cr*_ was reached. The average PNIPAM-co-GMA matrix height below the LCST is denoted as *h*_1_, whereas its counterpart above the LCST is denoted as *h*_2_. In all cases, an increase in cross-linking fraction, ν_*cr*_, leads to a decrease in the swelling ratio, *h*_1_ */h*_2_, and the effect is more significant at lower grafting density ρ_*g*_, (Figure 5).

**Figure 5:**
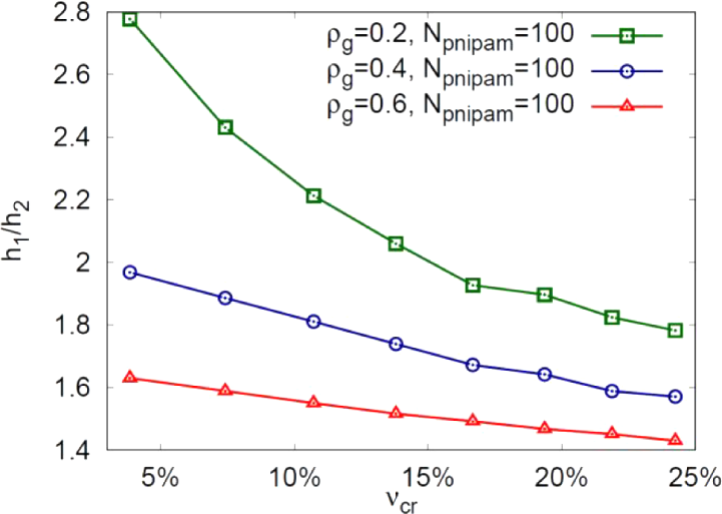
Effect of the cross-link fraction, ν_*cr*_, on the swelling ratio *h*_1_ */h*_2_, i.e., between the PNIPAM-co-GMA matrix height below and above LCST. Three different surface grafting densities, ρ_*g*_ = 0.2, 0.4, and 0.6, were analyzed.

The parameters of the model, *N*_*pnipam*_ = 100, ρ_*g*_ = 0.2, and a cross-link fraction in a range of ν_*cr*_ = 5–7%, lead to a swelling ratio of about 2.4–2.8, which is in accord with the experiments (Table 1).

Another problem relates to grafting the PNIPAM-co-GMA matrix to PGMA domains via reactive epoxy groups in both polymers. Such grafting can potentially restrict the swelling of PNIPAM-co-GMA. The DPD method was applied to perform simulations for microstructured surfaces with the geometry of a spherical cap (Figure 4) obtained in the experiments. The PGMA solid domains in our model were considered as two spherical caps on opposite sides of the simulation box. In between the domains is the substrate covered by PNIPAM-co-GMA. We considered a narrow simulation box, so the curvature of the spherical caps along the width is not significant; this allows us to save computational resources and also focus on a smaller set of parameters. The PNIPAM can be pinned to the substrate, and the domains have an independent density of pinning points. The pinning density to the substrate was fixed at ρ_g_ = 0.2, and for the domains, three possibilities ρ_pd_ = 0.2, 0.4, and 0.6 were considered. The radii of the spherical caps were *R* = 30, 50, and 100, and in each of these cases, the spherical cap height was 10, while the maximal height where PNIPAM-co-GMA can be pinned was also kept fixed as 7. The separation between domains, s, the distance between the nearest points on the domains at the level of the substrate, was considered as s = 10, 20, 30, and 40. In Figure 6, we show instantaneous frames from the simulation for the case *R* = 100, s = 40. Other parameters of the model can be found in the Supporting Information.

**Figure 6:**
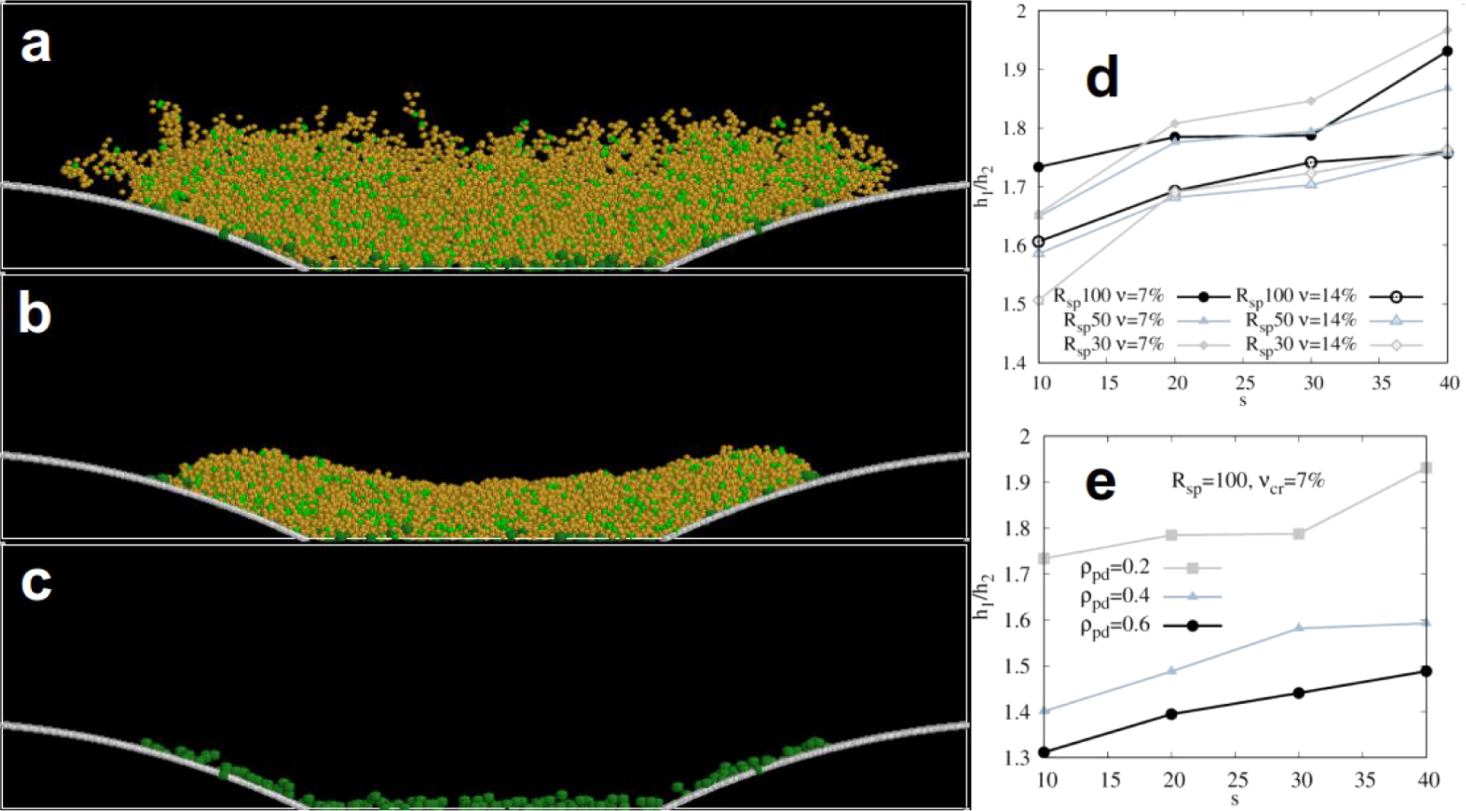
Simulation box with spherical domain caps and cross-linked PNIPAM-co-GMA matrix pinned to the substrate and domains (*R* = 100 and s = 40): (a) *T* < LCST, (b) *T* > LCST). The yellow beads represent PNIPAM; light green beads are cross-linking GMA points; and dark green beads are pinned points; (c) only pinning points are shown; (d) dependence of swelling on separation distance, s, for different *R* values; (e) dependence of swelling on separation distance, s, for different surface grafting densities, ρ_pd_. Note that the results in (d) and (e) are not for the fully equilibrated network; see the Supporting Information.

The results of the simulations are listed in Figure 6. There is little variation in the swelling ratio h_1_/h_2_ for the different radii (Figure 6d). The swelling ratio h_1_/h_2_ increases with the increment of separation distance (Figure 6e). An increase in the cross-link fraction ν_cr_ from 7% to 14% leads to a lower swelling ratio but does not affect the qualitative behavior significantly. On the contrary, the swelling ratio notably decreases when the pinning density on the domains, ρ_pd_, increases from 0.2 to 0.6. This effect is more significant than any considered variations in radii or cross-linking fraction.

Before discussing cell sorting on microstructured surfaces, we can draw the major conclusions from the characteristics of the microstructured surfaces: (1) a relatively narrow distribution of spacing between PGMA domains, which can be adjusted by the concentration of solutions used for spin coating; and (2) a broad distribution in the height of PGMA domains, which is important to consider when analyzing cell sorting experiments; (3) a narrow distribution of the PNIPAM matrix height; (4) the size of the PGMA domains and the surface coverage available for cell binding to the PGMA domains increase with the PGMA fraction in spin coating solutions in Step1; (5) the height of the PNIPAM-co-GMA domains in A1, B1, and C1 samples at *T* < LCST is very close to the height of PGMA domains at *T* > LCST (a low POF is expected), while for A2, B2, and C2 samples, the structure is substantially different and a high POF is expected; and (6) the PNIPAM-co-GMA matrix swelling is less dependent on domain geometry but is influenced by matrix cross-linking density, surface grafting density, and distance between domains.

### 3.5 Cell Sorting: Monte Carlo Simulations

We performed Monte Carlo computer simulations for the above-described model of the binary mixture of cell types 1 and 2 on four different micropatterned surfaces, which differ in PGMA domain size (Figure S21 and S22). We considered four values of the domain diameter, *D*_*d*_ *=* 2*R*_*d*_ (Figure 4) in reduced units (*D*_d_ = 0.5, 1.0, 1.5, and 2.0), while the coverage fraction of the surface by the PGMA domains was fixed and equal to σ_d_ = 0.48. Cells 1 and 2 differ only by the strength of attraction to the domains, as determined by the adhesion parameters *A*_1d_ = −0.4 and *A*_2d_ = −0.3, respectively (Table S4).

For each surface, simulations were performed in two sequential stages: cell adsorption and desorption. We considered the equivalent number of cells of both types in the system, equal to *N*_1_ = 100 and *N*_2_ = 100. At the beginning of the first stage, cells were randomly distributed within the simulation box and subsequently allowed to move stochastically, adhering to the domains upon encountering the surface (Figure S23 and S24). This occurred when the parameter of repulsive interaction of a cell with the polymer phase was *B*_p_ = 0.0, i.e., the PNIPAM-co-GMA was in the collapsed state and did not repel the cells. The adsorption stage consisted of 2 M simulation steps; however, adsorption is typically completed within 200–500 K steps. To ensure that the system had reached a stationary regime, the potential energy of the cells was monitored to verify its saturation. As a result, all cells were adsorbed onto the surface (*B*_p_ = 0.0), forming a monolayer with a surface density of ρ_c_ = (N_1_ + N_2_)/L^2^ = 3.472 × 10^–3^, corresponding to a surface packing fraction of 0.273. This indicates that cell crowding at the surface was negligible, and competition for available adhesion sites was minimal. A movie generated from our computer simulation illustrating the typical adsorption stage can be found in Supporting Information Video 1. All snapshots and movies obtained from our simulations were created with the help of the OVITO software.

In the second stage, the final configurations obtained during the first stage were used to initiate the desorption process by applying a repulsive field induced by the swollen polymer phase. This was achieved by varying the parameter *B*_p_. The desorption stage was conducted for 2 M simulation steps, although the system typically reached a stationary regime much earlier. In Figures S25 and S26, we demonstrate snapshots for the case of the domain sizes *D*_d_ = 0.5 and 2.0, respectively. It is clearly observed that an increase in *B*_p_ results in greater cell detachment. Moreover, a higher number of type 1 cells remains at the surface, while type 2 cells predominate in the environment. A movie generated from our computer simulation illustrating the typical desorption stage can be found in Supporting Information Video 2. It can also be noticed that cell detachment is more pronounced in the case of the smaller domains; however, a quantitative analysis is required to confirm this observation, which is presented below.

Based on the cell trajectories obtained from our simulations, the density profiles for both cell types were calculated and averaged over 1 M steps. Using these density profiles, the average number of cells located within a cutoff distance *r*_cut_ = D_1_/2 (or *D*_2_/2) from the surface was estimated for various values of *D*_d_ and *B*_p_. This approach allowed us to assess the efficiency of cell sorting under different conditions. In Figure 7a, we present the fractions of cells remaining attached to the surface, expressed as percentages. It can be seen that both types of cells undergo detachment as *B*_p_ increases. However, for type 1 cells, detachment is less pronounced and occurs with a significant delay along the *B*_p_ compared to type 2 cells, resulting in cell separation. The efficiency of this separation can be assessed using the separation factor, which reflects the relative amounts of the two cell types attached to the surface vs the initial adsorption ratio 1:1. We present this factor in Figure 7b as a function of the parameter *B*_p_, exhibiting a nonmonotonic dependence.

**Figure 7:**
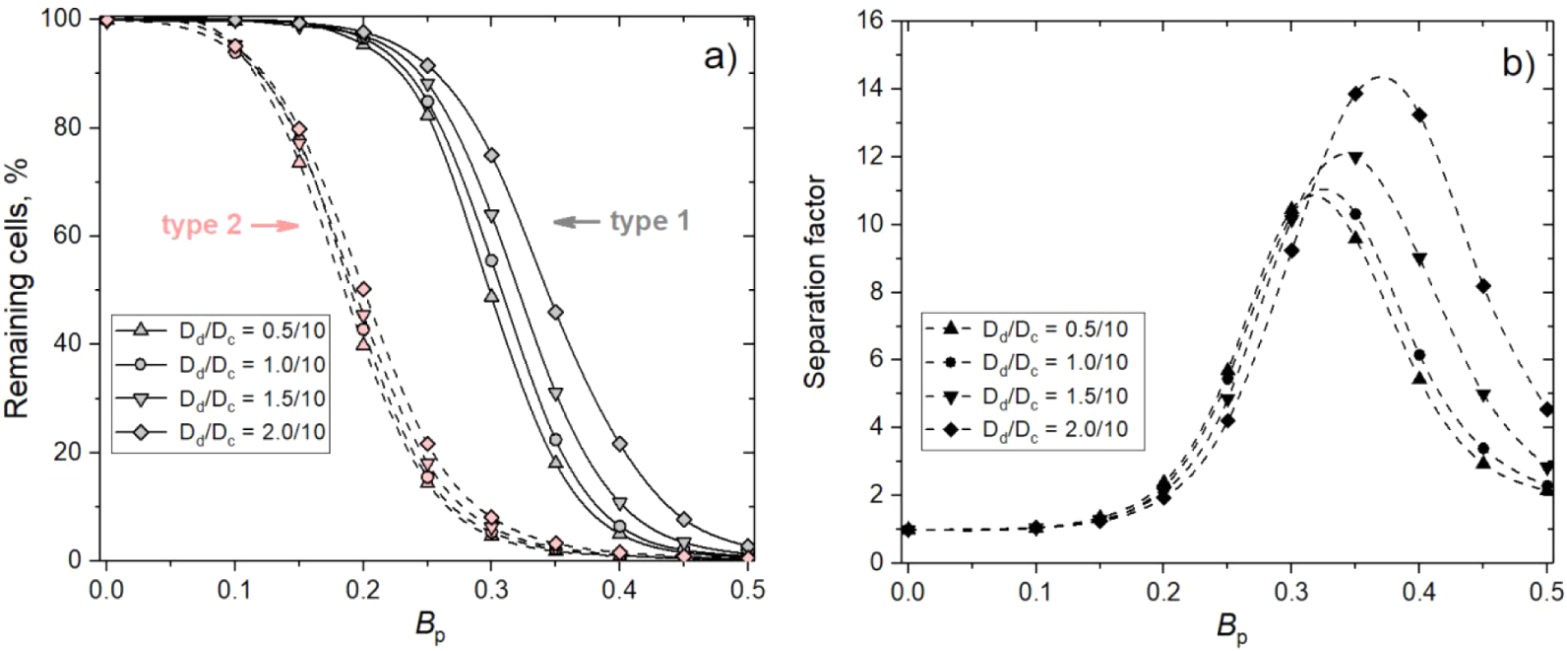
Results of computer simulations: (a) remaining cells on the surface and (b) separation factor simulation data as a function of the PNIPAM-co-GMA polymer-cell repulsion parameter *B*_p_ for different PGMA domain diameters *D*_d_ = 0.5, 1.0, 1.5, and 2 at a surface domain coverage of σ_d_ = 0.48. Adhesion parameters of cells of type 1 and 2 are equal to *A*_1*d*_ = −0.4 and *A*_2*d*_ = −0.3, respectively.

Assuming that a higher separation factor corresponds to better separation efficiency, the maxima of the curves obtained for different sizes of adhesive domains should indicate the values of *B*_p_, at which optimal separation performance is achieved. Interestingly, for larger domains, these maxima are higher than those for smaller domains and occur at higher values of *B*_p_. For instance, when the domain size is *D*_d_ = 0.5, the maximum occurs around *B*_p_ = 0.32, while for *D*_d_ = 2.0, it is around *B*_p_ = 0.37. It is worth noting that for *B*_p_ = 0.32 and *D*_d_ = 0.5, about 35% of type 1 cells and nearly 3% of type 2 cells remain attached to the surface. Approximately the same 35% of type 1 cells can be found for *D*_d_ = 2.0 at *B*_p_ = 0.37, while the fraction of type 2 cells is slightly lower (∼ 2.5%) than for *D*_d_ = 2.0 at *B*_p_ = 0.32. Nevertheless, this small difference is sufficient to increase the separation factor by about 20% when the domain size is increased from *D*_d_ = 0.5 to *D*_d_ = 2.0. It should be noted that this effect is analyzed under the fixed coverage fraction of the surface by the PGMA domains. The interplay between the domain size and surface coverage remains a promising direction for future research.

To summarize, the choice of domain size plays an important role in cell sorting and should be taken into account, even when the coverage fraction remains the same. We found that increasing the domain size can enhance the efficiency of cell sorting. Another important parameter—the repulsion strength or POF, which depends on the polymer phase density—must be carefully adjusted to achieve a fine balance with the adhesive properties of the domains, domain sizes, and the coverage fraction. The model developed in this study, based on Monte Carlo simulations, can assist in identifying consistent and reasonable values for these parameters.

Microstructures with larger PGMA domains exhibit higher sorting efficiency; however, the outcome also depends on the strength of the repulsive force exerted by the polymer phase. If the repulsion is too weak or too strong, the sorting process becomes ineffective.

### 3.6 Cell Sorting Experiments

The fabricated microstructured surfaces were tested in a series of experiments that can be divided into two groups. In one group, cells were seeded on the surface and incubated for a “short” time of 20 min, while in another group, the incubation time was “long” for 16 h. For HaCaT cells, a 20 min incubation time was not sufficient to bind cells to the surface, so we increased the incubation time to 1 h instead of 20 min. These differences probe cell sorting at different stages of cell adhesion. In each group, we tested microstructured surfaces for the adhesion and detachment of individual cells and their mixtures.

The cells were seeded at 37 °C, and after incubation in a CO_2_ incubator, they were visualized on the surface at 37 °C and after cooling down to room temperature *T* < LCST. One-component PGMA and PNIPAM-co-GMA films were used as controls. The poorly bound cells were suspended using either gentle pipetting or directed media flow using a peristaltic pump (PP). Pipetting is broadly used to collect loosely attached cells, but even gentle pipetting can develop a noticeable shear force acting on the cells. This force adds to the POF. The impact of the shear flow was minimized by using a media flow of about 4 mL/min generated by the peristaltic pump flow (Figure S10).

We used 3T3, HaCaT, and RAW cells, which are well-known for their different adhesive properties. RAW cells are more adhesive to standard cell culture materials (plasma-treated polystyrene dishes) compared to 3T3 and HaCaT cells. Representative images for 20 min and 16 h incubation times for a 3T3 and RAW cell mixture on the microstructured surface A1 are shown in Figures 8 and 9. For both incubation times, cells strongly adhered to PGMA control surfaces and did not detach at *T* < LCST, whereas the cells did not attach well to PNIPAM-co-GMA control coatings at 37 °C.

**Figure 8:**
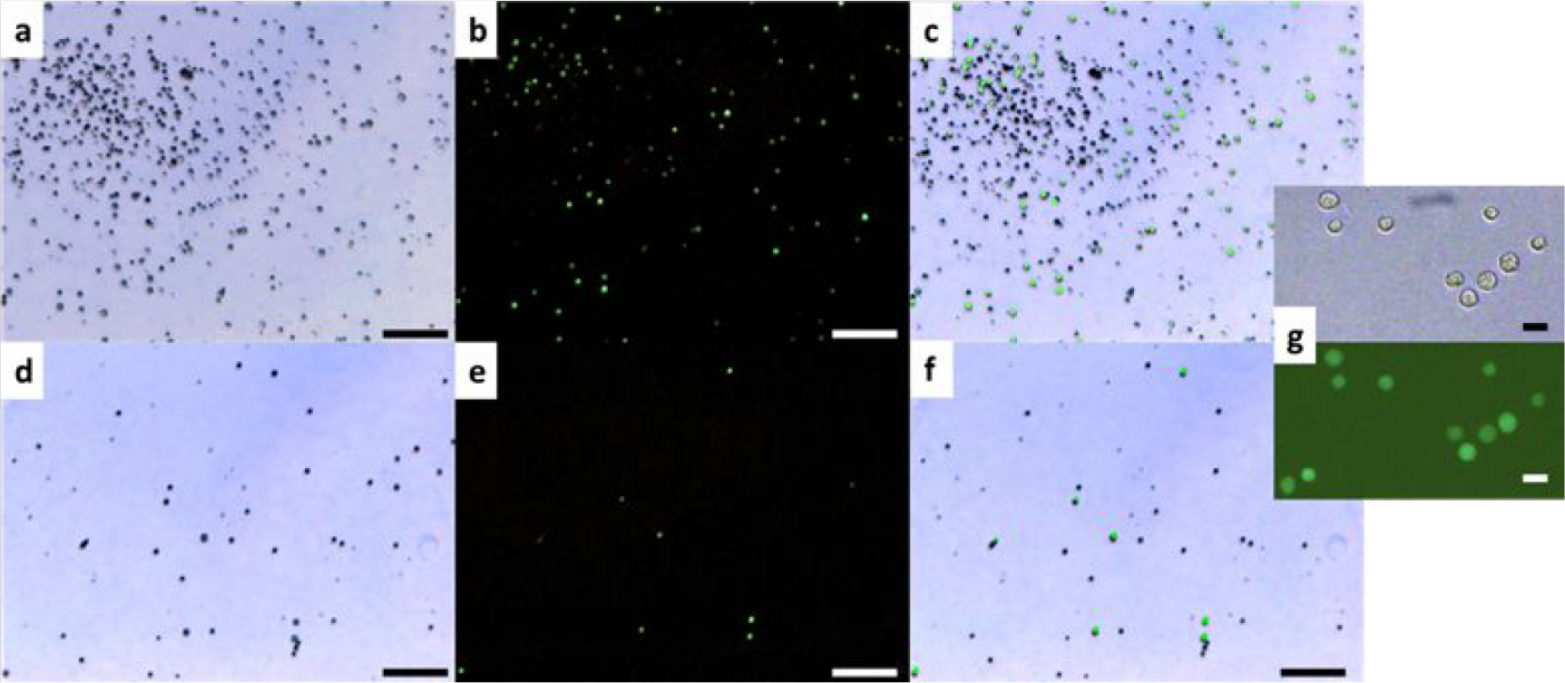
Optical images of a mixture of RAW and 3T3 cells on the microstructured A1 surface: (a–c) after 20 min incubation at 37 °C (*T* > LCST) and (d–f) after cooling to room temperature (*T* < LCST) and pipetting: (a,d) no fluorescent filter applied; (b,e) fluorescent filter applied to visualize only 3T3 cells; (c,f) overlay of (a) and (b), and (d) and (e) images, respectively; and (g) zoomed up images of 3T3 cells on the microstructured surface after 20 min incubation. (a–f) Scale bars are 200 μm and (g) scale bar is 20 μm.

**Figure 9:**
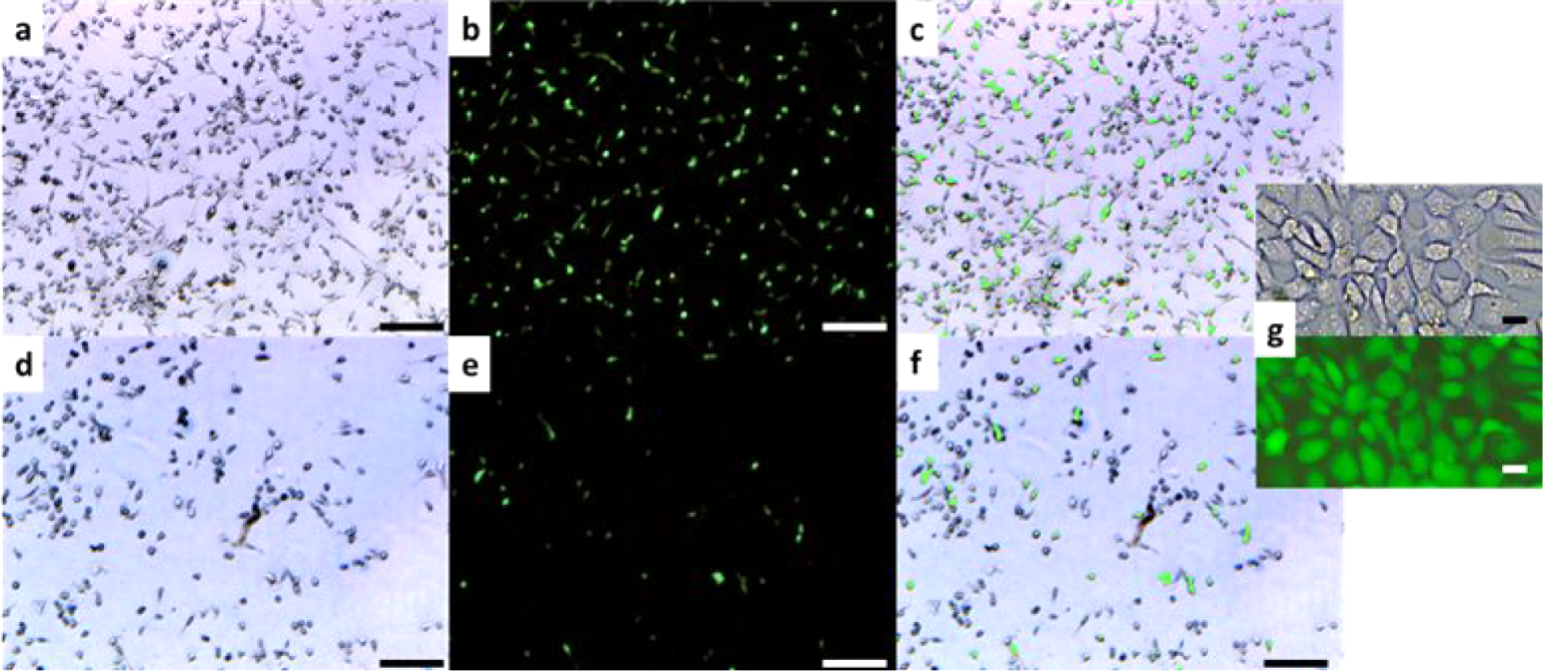
Optical images of a mixture of RAW and 3T3 cells on the microstructured A1 surface: (a–c) after 16 h of incubation at 37 °C (*T* > LCST) and (d–f) after cooling to room temperature (*T* < LCST) and pipetting: (a,d) no fluorescent filter applied; (b,e) fluorescent filter applied to visualize only 3T3 cells; (c,f) overlay of (a) and (b), and (d) and (e) images, respectively; and (g) zoomed up images of 3T3 cells on the microstructured surface after 16 h of incubation. (a–f) Scale bars are 200 μm and (g) scale bar is 20 μm.

From Figures 8 and 9, it is evident that 3T3 cells adhere more weakly to the microstructured surface than RAW cells. The number of cells that adhere to the surface under *T* > LCST conditions at 20 min is significantly less than the number of cells observed after 16 h. After 20 min, the cells are weakly bound, and their shape remains unchanged (Figure 8g). After 16 h, the cells are strongly bound and elongated (Figure 9g). The increased amount is due to cell division on the surface. For both incubation times, after cooling to *T* < LCST, a high fraction of 3T3 cells detached from the surface. For the quantitative evaluation of the sorting efficiency, we used the separation factor (SF) and the percentage of the total number of remaining cells (both types of cells). SF= N_1i_/N_2i_:N_1s_/N_2s_, where N_1i_ and N_2i_ are the initial numbers of cell types 1 and 2 in the mixture after binding to the surface, N_1s_ and N_2s_ are the numbers of cell types 1 and 2 on the surface after separation (cooling). The ratios of RAW:3T3 cells prior to cooling and after cooling were obtained using image analysis, and the results are shown in Figure 10 (using pipetting) and Figure 11 (used PP flow).

**Figure 10:**
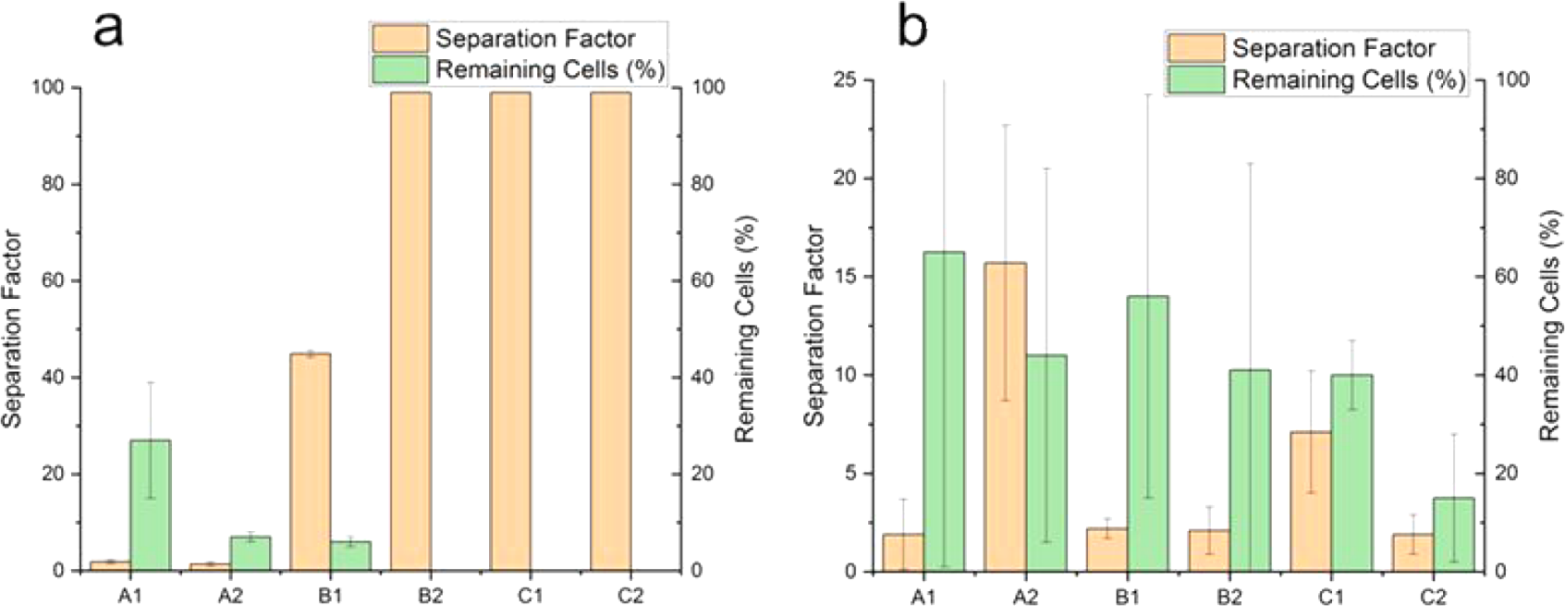
Sorting RAW:3T3 mixtures after (a) 20 min and (b) 16 h of incubation; pipetting. The separation factor (orange) and remaining cell percentages (green) are shown following pipetting and detachment at room temperature (*T* < LCST) from A1–C2 surfaces.

**Figure 11:**
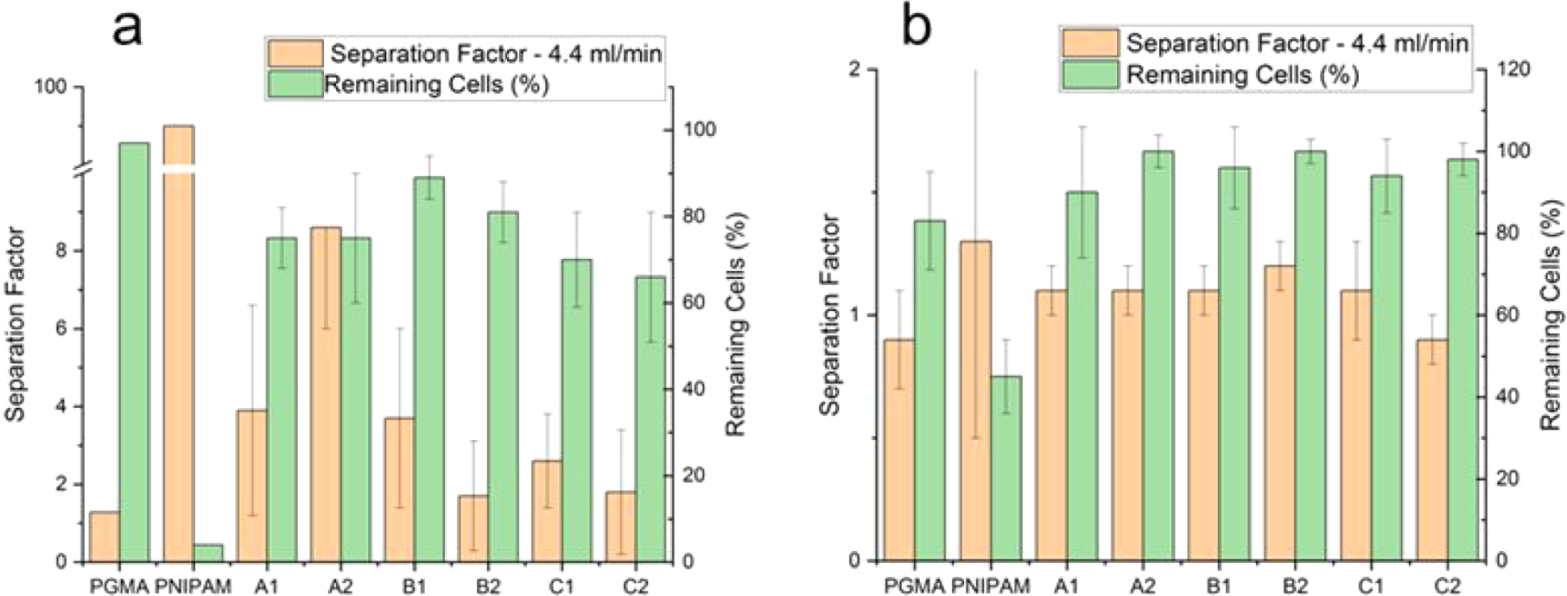
Sorting RAW:3T3 mixtures after (a) 20 min and (b) 16 h of incubation; PP flow. The separation factor (orange) and remaining cell percentage (green) are shown following PP (4 mL/min) and detachment at room temperature (*T* < LCST) from A1–C2 surfaces.

The results for the control surfaces, namely, single-component PGMA and PNIPAM, are not shown in Figure 10 because all seeded cells remained on the PGMA surface, and all cells were removed by pipetting from the PNIPAM-co-GMA surface, resulting in a separation factor of 1. For microstructured surfaces, we observed separation factors greater than 1 for both incubation times, but the sorting efficiency was greater for the 20 min incubation time (weaker cell binding).

With a decrease in the PGMA:PNIPAM-*co*-GMA ratio, A > B > C samples (less PGMA used), the PGMA domains become smaller, leading to a reduction in the surface coverage by PGMA domains for binding cells (Table 1). Hence, the weakly adhered 3T3 cells are easily removed by the POF. Consequently, a high RAW:3T3 ratio on the surface is achieved (Figure 10a), demonstrating not only effective sorting, with a separation factor of 99, but also causing the surfaces to be so repellent that a very limited number of remaining cells was observed, although those that remained were nearly exclusively RAW cells. This effect is exacerbated when A2, B2, and C2 samples are used in comparison to A1, B1, and C1, as PNIPAM-co-GMA swelling (*T* < LCST) substantially exceeds the height of the PGMA domains (high POF). The swollen PNIPAM-co-GMA matrix exceeds the PGMA domains in A2, B2, and C2 samples by a much greater distance than the 25 nm integrin binding distance. The most extreme case was observed for C2 samples when, after 16 h of incubation, < 20% of cells remained on the surface, with a low separation factor of about 2 (Figure 10b).

The data from the experiments with the same cell cultures and microstructured surfaces, but with a controlled PP shear flow, are listed in Figure 11. Manual pipetting is not well controlled, and hence, it is difficult to reproduce the shear force added to POF. As an indirect measure of the shear force, we used the media flux through the needle. We applied an empirically selected flow rate of 4 mL/min. The observed increased amount of remaining cells on the surfaces proves that using controlled flux vs pipetting provides less added shear force. The results also support the prior statement that short-term weak adhesion favors sorting. The reference data (control samples) with one-component PGMA and PNIPAM-co-GMA surfaces are shown in Figure 11. After 20 min of incubation time, a large fraction of cells remained on the PGMA surface, resulting in no separation, while almost all cells were detached from the PNIPAM-co-GMA surface (Figure 11a). This effect is significantly diminished in Figure 11b for 16 h incubation time, with PGMA showing a remaining cell percentage of 87% and PNIPAM-co-GMA having 45%, thus illustrating the lessened difference between the control samples, which can only be attributed to cell binding and increased adhesion to the surface. However, no sorting was achieved with these surfaces or any microstructured surfaces after 16 h of incubation (Figure 11b). All the samples demonstrate sorting factors (about 1) similar to the single-component PGMA surface.

The effect of the microstructured surfaces is similarly easily identifiable during the shorter 20-min incubation period, with A1 and B1 having a separation factor of approximately 4, and A2 having a separation factor of 8. For all microstructured surfaces, the total percentage of cells remaining on the surface exceeds 60%, with B1 and B2 being >80%. The best sorting result was obtained for A2. The reason for this is the large surface areas of the A samples’ PGMA domains compared to B and C. Similar to pipetting results, it is clear that a closer domain-to-matrix height is the most effective, with the high PGMA domains offering sufficient binding capacity for the cells, while the higher PNIPAM-co-GMA thickness is equally more effective at swelling to and past the domains to detach cells and sort effectively. Another important observation is the effect of the shear flow. From a comparison of Figures 10 and 11, we can conclude that the drop in shear force (for PP flow) shifted the efficiency of cell sorting from C samples to A samples. This is in accord with the analysis of the effect of the thickness of PNIPAM-co-GMA vs PGMA domains. The stronger combined (swollen matrix plus the shear flow) POF becomes less efficient above some threshold, proving our statement that efficient sorting occurs for the optimally adjusted POF in a range AdA < POF < AdB.

In our experiments, we did not evaluate cell adhesion quantitatively to establish a correlation between cell adhesion and sorting efficiency, which can be a future development of this research. However, we used an arbitrary evaluation of cell adhesion for three different cell cultures on the surfaces used in this study. This arbitrary evaluation is based on observations made during work with the cell cultures. Based on such observations, the cells can be arranged according to adhesion strength: RAW > HaCaT > 3T3. HaCaT cells need longer initial “short” incubation times compared to 3T3 cells, with most cells not attaching at all after 20 min; however, following 1 h, reproducible binding results were obtained, with HaCaT cells having slightly better binding than 3T3 cells, as shown in Figure 12. Surprisingly, such a dependence does not directly correlate with the time course of trypsin/EDTA treatment required for complete cellular detachment from plasma-treated polystyrene plasticware. Usually, the trypsin/EDTA treatment time for NIH-3T3 cells is about 2.5–3 min, while for HaCaT cells, such treatment time is about 13–15 min.

**Figure 12:**
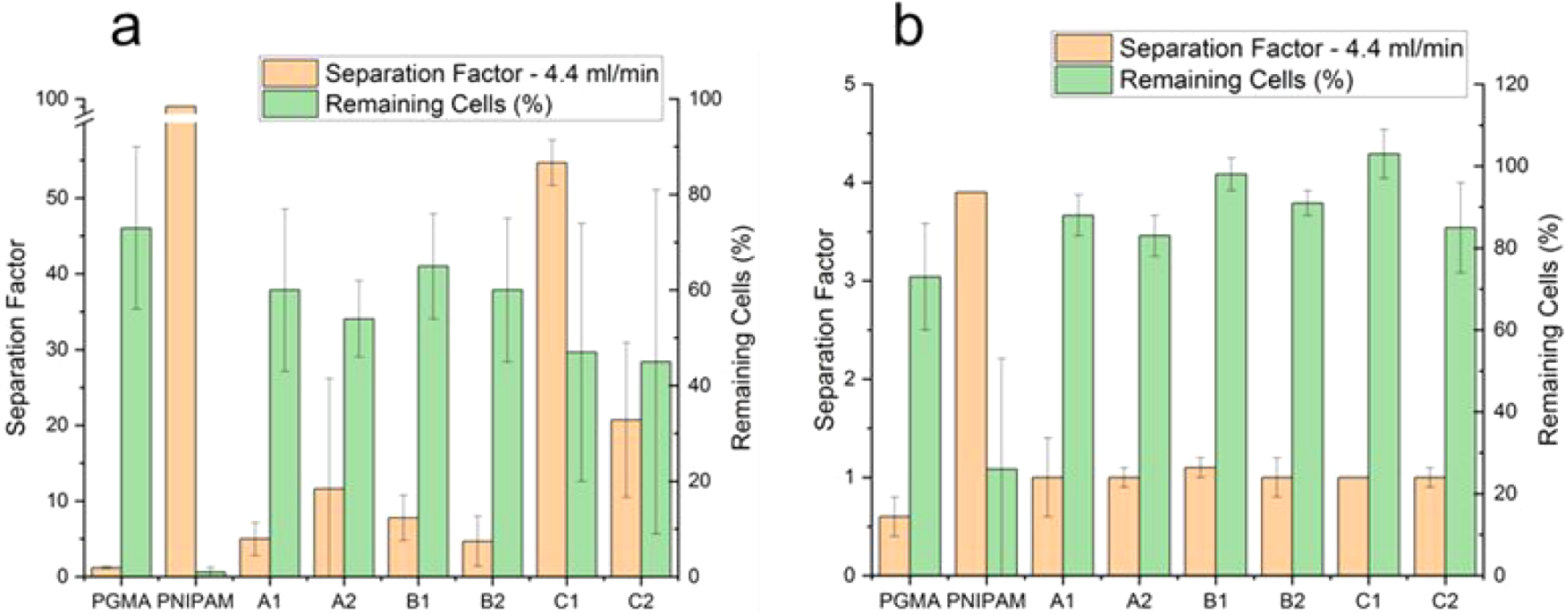
Sorting RAW:HaCaT mixtures after (a) 1 h and (b) 16 h of incubation; PP flow. The separation factor (orange) and remaining cell percentage (green) are shown following PP (4 mL/min) and detachment at room temperature (*T* < LCST) from A1–C2 surfaces.

As can be seen in Figure 12a, the reference surfaces of PGMA and PNIPAM-co-GMA show opposite effects on the separation and removal of cells, illustrated by the percentage of cells that remained, with PGMA having the highest of all surfaces at 73%, which is to be expected, while PNIPAM-co-GMA has almost zero cells remaining, which is also expected. The separation factor for PGMA is expected to be near 1, indicating insignificant levels of cell sorting. The microstructured surfaces, on the contrary, show vastly significant separation factors for RAW/HaCaT mixtures, with minimum values ranging from 5 to 12 for A1, A2, B1, and B2, while C1 and C2 have separation values of 54.7 and 20.7, respectively, which represents a very high selection bias, especially considering the number of cells remaining is near 50% in all cases. Similar to RAW:3T3 sorting, however, once cells have been incubated for 16 h, the effect of the surface structure becomes much less pronounced. Separation is thus again achieved, following short incubation periods.

The most difficult task was to sort 3T3 and HaCaT cells. Both cell types are less adherent, and it would be more significant to be able to sort these cells from one another. HaCaT requires 1 h to adhere to surfaces; thus, this time served as the minimum incubation time for both cell types in a mixture, while 16 h was still considered the long incubation period. From Figure 13, it is clear that sorting the cells was not as successful as when either was sorted from RAW cells. Figure 13 shows data reminiscent of both prior sorting experiments when considering the PGMA and PNIPAM-co-GMA reference surfaces, with separation factor values of about 1 and nearly 100% of the remaining cells following sorting for PGMA, while PNIPAM-co-GMA showed excellent cell removal and no separation factor data being calculable from the insignificant number of cells. The microstructured surfaces, however, show a linear SF result regardless of the domains or matrix characteristics, with the only variation being minute at best. The remaining cell percentages in Figure 13a vary to some degree; however, in Figure 13b, the variation is negligible. An untested alternative to the experimental results shown in Figure 13 is whether an incubation time of less than 1 h was used. This would have prevented HaCaT attachment and made sorting much more likely. This opens multiple avenues for cell sorting using an identical set of surfaces and can be the basis for future research. The collection of the optical images for the cell sorting experiments can be requested from the corresponding author, and the results of the image processing are in Table S3.

**Figure 13:**
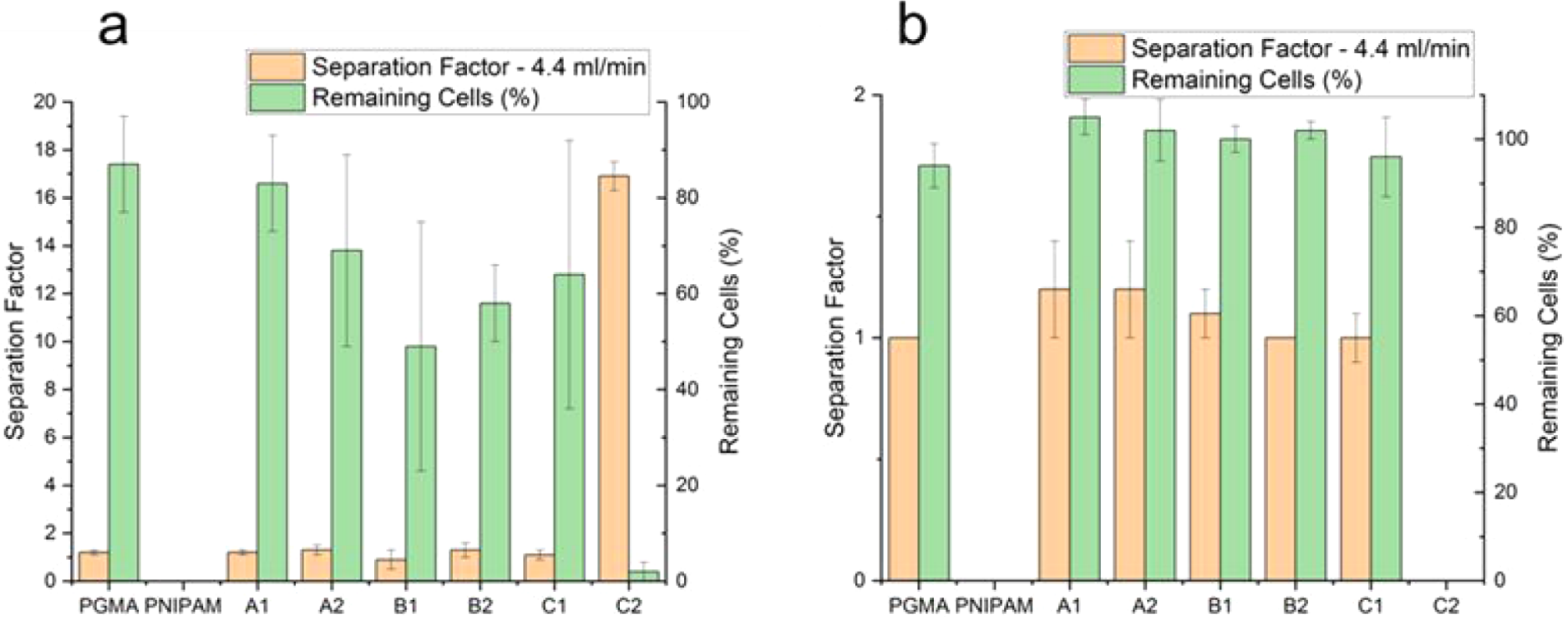
Sorting HaCaT:3T3 mixtures after (a) 1 h and (b) 16 h of incubation (PP flow). The separation factor (orange) and remaining cell percentage (green) are shown following PP (4 mL/min) and detachment at room temperature (*T* < LCST) from A1–C2 surfaces.

## 4 Conclusions

The experiments and computer simulations provide solid evidence for the feasibility of label-free cell sorting based on weak nonspecific interactions with dynamic stimuli-responsive microstructured surfaces. The separation mechanism, based on the adsorption-detachment of cells on and from the microstructured surfaces, resembles the chromatography of small molecules, where separation is possible within the optimal range of molecule-adsorbent interactions. However, the separation of very weakly and very strongly interacting molecules with the adsorbent is not efficient. In other words, if *kT* is used as a measure of the interaction strength of small molecules with the adsorbent, separation becomes impossible if the adsorption energy is very much lower or greater than *kT*. In the latter cases, a change in the adsorbent and temperature is used to improve separation. The push-off force on the stimuli-responsive surface plays a similar role for cells as thermal fluctuations do for small molecules. The adhesive, PGMA, domains provide cell binding, while thermoresponsive PNIPAM-*co*-GMA domains push cells off at *T* < LCST. This combination, if appropriately adjusted so that the push-off force is between the adhesive forces of weakly and strongly bound cells AdA < POF < AdB, cell separation can be very efficient in terms of separation factor and the number of separated cells.

The balance between cell binding and detachment forces is approached by adjusting the surface structure. For the given chemical structure of the adhesive domains, adhesion can be adjusted by changes in the contact area or surface coverage of the adhesive domains. The kinetics of cell binding are also important. Cell adhesion increases with time. Push-off force can also be adjusted by many factors related to the properties of stimuli-responsive domains: surface coverage, swelling ratio, cross-linking, and the ratio of heights of adhesive and stimuli-responsive domains.

In this work, we successfully separated cells that were substantially different in their adhesive properties from the studied microstructured thermoresponsive surfaces, while the separation of cells with closer adhesive behavior has not yet been successful (a very low separation factor). This problem can be approached by optimizing the geometry of the microstructured surface or using a multistep separation process, and it can be the subject of future research.

Along with the separation mechanism, we also demonstrate a simple method for the fabrication of microstructured thermoresponsive surfaces based on the phase separation of PGMA and PNIPAM-co-GMA copolymers in thin films. The microstructure at the submicrometer level can be regulated by the ratio of the two polymers. The GMA functional groups are used to cross-link the film and graft it to the substrate. The swelling ratio of the thermoresponsive domain is regulated by the cross-link density, surface grafting density, and geometry of the microstructures. The best combination of these parameters for cell sorting can be predicted with mesoscale computer simulations, which have already demonstrated their potential in this study. The developed materials can find applications in scalable and cost-efficient cell sorting technologies.

## Supplementary Material







## Notes

Schematics of experiments, characteristics of the polymers and microstructured surfaces, details of models used in computer simulations, and cell sorting optical images (PDF)
Monte Carlo simulation of cell adsorption (MP4)
Monte Carlo simulation of cell detachment (MP4)

## References

[1] Fulwyler M. J. (1965). Electronic
Separation of Biological Cells by Volume. Science.

[2] Plouffe B. D., Murthy S. K., Lewis L. H. (2015). Fundamentals and Application of Magnetic
Particles in Cell Isolation and Enrichment: A Review. Rep. Prog. Phys..

[3] Sutermaster B. A., Darling E. M. (2019). Considerations for High-Yield, High-Throughput Cell
Enrichment: Fluorescence Versus Magnetic Sorting. Sci. Rep..

[4] Qiu X., Lombardo J. A., Westerhof T. M., Pennell M., Ng A., Alshetaiwi H., Luna B. M., Nelson E. L., Kessenbrock K., Hui E. E., Haun J. B. (2018). Microfluidic Filter Device with Nylon
Mesh Membranes Efficiently Dissociates Cell Aggregates and Digested
Tissue into Single Cells. Lab Chip..

[5] Wu Y., Ren Y., Tao Y., Hou L., Jiang H. (2018). High-Throughput Separation,
Trapping, and Manipulation of Single Cells and Particles by Combined
Dielectrophoresis at a Bipolar Electrode Array. Anal. Chem..

[6] Lu H., Gaudet S., Schmidt M. A., Jensen K. F. (2004). A Microfabricated
Device for Subcellular Organelle Sorting. Anal.
Chem..

[7] Du M., Kavanagh D., Kalia N., Zhang Z. (2019). Characterising the
Mechanical Properties of Haematopoietic and Mesenchymal Stem Cells
Using Micromanipulation and Atomic Force Microscopy. Med. Eng. Phys..

[8] Sims N. R., Anderson M. F. (2008). Isolation of Mitochondria
from Rat Brain Using Percoll
Density Gradient Centrifugation. Nat. Protoc..

[9] Khare, P. ; Pant, A. Cell Sorting: Underpinnings and Contemporary Developments. In Flow Cytometry: Applications in Cellular and Molecular Toxicology; Springer, 2025; pp. 1–13.

[10] Gossett D.
R., Weaver W. M., Mach A. J., Hur S. C., Tse H. T. K., Lee W., Amini H., Di Carlo D. (2010). Label-Free Cell Separation
and Sorting in Microfluidic Systems. Anal. Bioanal.L
Chem..

[11] Shiri, F. ; Feng, H. ; Gale, B. K. Chapter 14 - Passive and Active Microfluidic Separation Methods. Particle Separation Techniques. Contado, C. pp. 449–484.Elsevier, 2022

[12] Du Z., Colls N., Cheng K. H., Vaughn M. W., Gollahon L. (2006). Microfluidic-Based
Diagnostics for Cervical Cancer Cells. Biosens.
Bioelectronics.

[13] Sin A., Murthy S. K., Revzin A., Tompkins R. G., Toner M. (2005). Enrichment
Using Antibody-Coated Microfluidic Chambers in Shear Flow: Model Mixtures
of Human Lymphocytes. Biotechnol. Bioeng..

[14] Chang W. C., Lee L. P., Liepmann D. (2005). Biomimetic
Technique for Adhesion-Based
Collection and Separation of Cells in a Microfluidic Channel. Lab Chip..

[15] Bose S., Singh R., Hanewich-Hollatz M., Shen C., Lee C.-H., Dorfman D. M., Karp J. M., Karnik R. (2013). Affinity Flow Fractionation
of Cells Via Transient Interactions with Asymmetric Molecular Patterns. Sci. Rep..

[16] Choi S., Karp J. M., Karnik R. (2012). Cell Sorting
by Deterministic Cell
Rolling. Lab Chip..

[17] Tasadduq B., McFarland B., Islam M., Alexeev A., Sarioglu A. F., Sulchek T. (2017). Continuous
Sorting of Cells Based on Differential P
Selectin Glycoprotein Ligand Expression Using Molecular Adhesion. Anal.L Chem..

[18] Chrit F. E., Li P., Sulchek T., Alexeev A. (2024). Adhesion-Based High-Throughput Label-Free
Cell Sorting Using Ridged Microfluidic Channels. Soft Matter..

[19] Kwon K. W., Choi S. S., Lee S. H., Kim B., Lee S. N., Park M. C., Kim P., Hwang S. Y., Suh K. Y. L.-F. (2007). Microfluidic
Separation and Enrichment of Human Breast Cancer Cells by Adhesion
Difference. Lab Chip..

[20] Sniadecki N. J., Desai R. A., Ruiz S. A., Chen C. S. (2006). Nanotechnology for
Cell–Substrate Interactions. Ann. Biomed.L
Eng..

[21] Lim J. Y., Hansen J. C., Siedlecki C. A., Runt J., Donahue H. J. (2005). Human Foetal
Osteoblastic Cell Response to Polymer-Demixed Nanotopographic Interfaces. J. Royal Soc. Interface.

[22] Green J. V., Murthy S. K. (2009). Microfluidic Enrichment
of a Target Cell Type from
a Heterogenous Suspension by Adhesion-Based Negative Selection. Lab Chip..

[23] Housmans C., Sferrazza M., Napolitano S. (2014). Kinetics of Irreversible Chain Adsorption. Macromolecules.

[24] O’Shaughnessy B., Vavylonis D. (2003). Irreversibility
and Polymer Adsorption. Phys. Rev. Lett..

[25] Yu C., Granick S. (2014). Revisiting Polymer
Surface Diffusion in the Extreme
Case of Strong Adsorption. Langmuir.

[26] Aveyard R., Binks B. P., Clint J. H. (2003). Emulsions
Stabilised Solely by Colloidal
Particles. Adv. Colloid Interface Sci..

[27] Nagrath S., Sequist L. V., Maheswaran S., Bell D. W., Irimia D., Ulkus L., Smith M. R., Kwak E. L., Digumarthy S., Muzikansky A., Ryan P., Balis U. J., Tompkins R. G., Haber D. A., Toner M. (2007). Isolation of Rare Circulating Tumour
Cells in Cancer Patients by Microchip Technology. Nature.

[28] Poulichet V., Garbin V. (2015). Ultrafast Desorption of Colloidal Particles from Fluid
Interfaces. Proc. Natl. Acad. Sci. U. S. A..

[29] Kurashina Y., Imashiro C., Hirano M., Kuribara T., Totani K., Ohnuma K., Friend J., Takemura K. (2019). Enzyme-Free Release
of Adhered Cells from Standard Culture Dishes Using Intermittent Ultrasonic
Traveling Waves. Commun. Biol..

[30] Garcia A. J., Gallant N. D. (2003). Stick and Grip: Measurement Systems
and Quantitative
Analyses of Integrin-Mediated Cell Adhesion Strength. Cell Biochem. Biophys..

[31] Murphy-Ullrich J. E. (2001). The De-Adhesive
Activity of Matricellular Proteins: Is Intermediate Cell Adhesion
an Adaptive State?. J. Clinic.Invest..

[32] Pierres A., Benoliel A.-M., Bongrand P. (2002). Cell Fitting
to Adhesive Surfaces:
A Prerequisite to Firm Attachment and Subsequent Events. Eur. Cell Mater..

[33] Anselme K., Ploux L., Ponche A. (2010). Cell/Material Interfaces: Influence
of Surface Chemistry and Surface Topography on Cell Adhesion. J. Adhesion Sci. Techn..

[34] Anselme K., Bigerelle M., Noel B., Dufresne E., Judas D., Iost A., Hardouin P. (2000). Qualitative and Quantitative Study
of Human Osteoblast Adhesion on Materials with Various Surface Roughnesses. J. Biomed. Mater. Res..

[35] Anselme K., Bigerelle M. (2006). Modelling
Approach in Cell/Material Interactions Studies. Biomaterials.

[36] Kim Y., Laradji A. M., Sharma S., Zhang W., Yadavalli N. S., Xie J., Popik V., Minko S. (2022). Refining of Particulates at Stimuli-Responsive
Interfaces: Label-Free Sorting and Isolation. Angew. Chem., Int. Ed..

[37] Yamada N., Okano T., Sakai H., Karikusa F., Sawasaki Y., Sakurai Y. (1990). Thermo-Responsive Polymeric Surfaces; Control of Attachment
and Detachment of Cultured Cells. Makromol.
Chem., Rapid Commun..

[38] Kim Y., Jahan U., Deltchev A., Lavrik N., Reukov V., Minko S. (2023). Strategy to Non-Enzymatic
Harvesting of Cells Via Decoupling of Adhesive
and Disjoining Domains of Nanostructured Stimuli-Responsive Polymer
Film. ACS Appl. Mater. Interfaces.

[39] Badenhorst R., Makaev S., Yaremchuk D., Sajjan Y., Sulimov A., Reukov V. V., Lavrik N. V., Ilnytskyi J., Minko S. (2024). Reversible Binding Interfaces Made
of Microstructured Polymer Brushes. Langmuir.

[40] Ilnytskyi J., Yaremchuk D., Minko S. (2025). Interaction of Colloidal Particulates
with Dynamic Microstructured Polymer Brushes: Computer Simulations. Langmuir.

[41] Tsien R. Y. (1998). The Green
Fluorescent Protein. Annu. Rev. Biochem..

[42] Chalfie M., Tu Y., Euskirchen G., Ward W. W., Prasher D. C. (1994). Green Fluorescent
Protein as a Marker for Gene Expression. Science.

[43] Cabantous S., Terwilliger T. C., Waldo G. S. (2005). Protein Tagging and Detection with
Engineered Self-Assembling Fragments of Green Fluorescent Protein. Nature Biotechn..

[44] Groot R. D., Warren P. B. (1997). Dissipative Particle
Dynamics: Bridging the Gap between
Atomistic and Mesoscopic Simulation. J. Chem.
Phys..

