# Supplementary material for: Dynamic Microstructured Thermoresponsive Interfaces for Label-Free Cell Sorting Based on Nonspecific Interactions
Source: ACS Appl Mater Interfaces. 2025 Aug 20;17(35):49193–209. doi: 10.1021/acsami.5c08747 (PMC12412105; doi:10.1021/acsami.5c08747)
Supplement: Supplementary file 1 [file am5c08747_si_001.pdf]

Supporting Information

Dynamic Microstructured Thermoresponsive Interfaces for Label-Free Cell Sorting Based on Nonspecific Interactions

Ronaldo Badenhorst[a], Sergei V. Makaev[a], Mikhail Parker[a], Rostyslav Marunych[b], Vladimir Reukov[c], Agnieszka Będzińska[b], Olexandr Korchynskyi[b], Ostap Kalyuzhnyi[d], Dmytro Yaremchuk[d,e], Jaroslav Ilnytskyi [d,e], Taras Patsahan[d,e], Sergiy Minko[a]*

[a] Nanostructured Materials Lab, University of Georgia, Athens, 30602 GA, United States

[b] Laboratory of Experimental Biology and Department of Biochemistry and General Chemistry, Medical Faculty, Rzeszów University; Warzywna 1a Str., 35-959, Rzeszów, Poland

[c] Regenerative Bioscience Center, Department of Textiles, Merchandising and Interiors, Athens, 30602 GA, United States

[d] Yukhnovskii Institute for Condensed Matter Physics of the National Academy of Sciences of Ukraine, 1 Svientsitskii str., 79011, Lviv, Ukraine

[e] Institute of Applied Mathematics and Fundamental Sciences, Lviv Polytechnic National University, 12 S. Bandera Str., 79013, Lviv, Ukraine

* Corresponding author e-mail: sminko@uga.edu

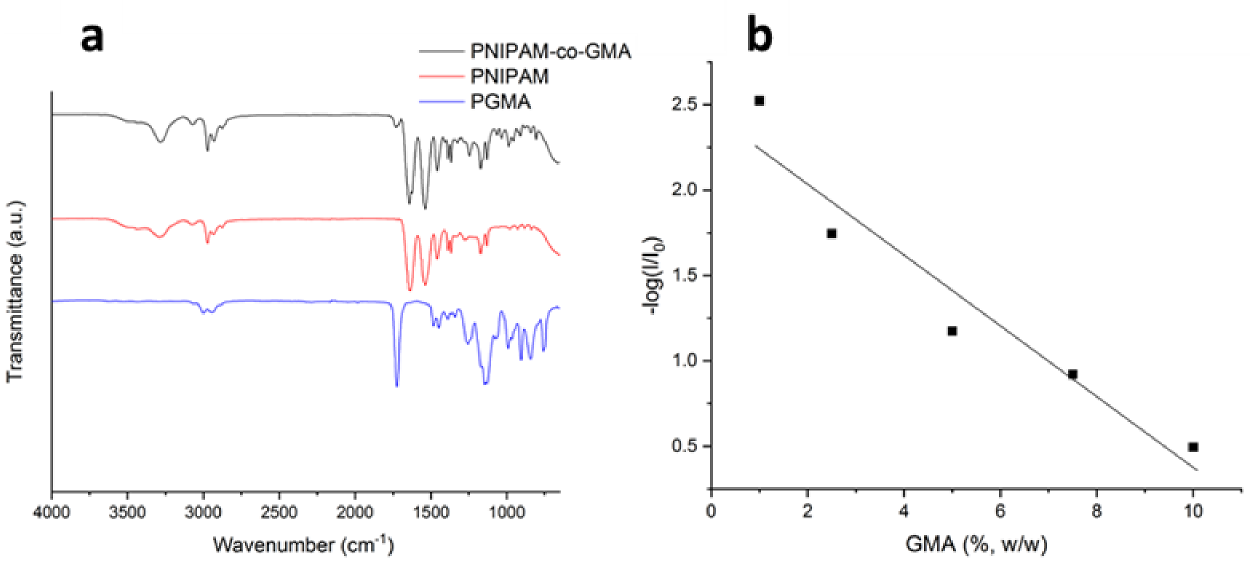

Figure S1. FTIR analysis of the PNIPAM-co-GMA copolymer. (a) spectra of PGMA, PNIPAM and PNIPAM-co-GMA. (b) calibration curve for intensities ratios at 1727 cm^{-1} (carbonyl GMA) and 1642 cm^{-1} (carbonyl NIPAM).

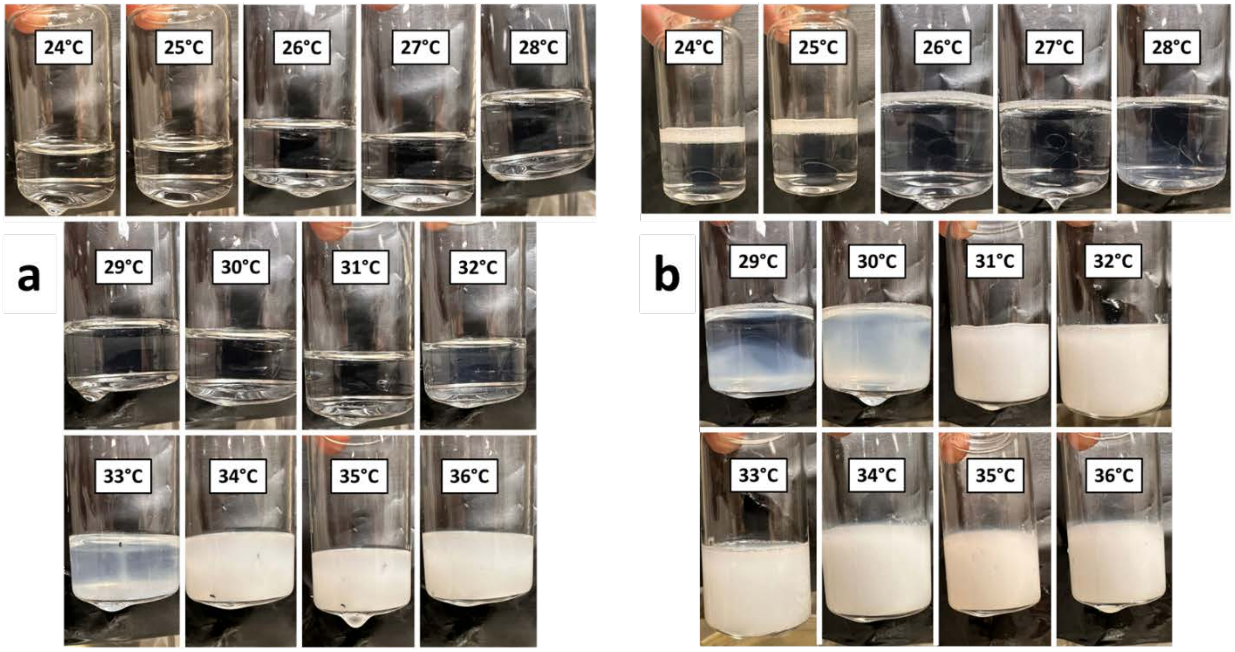

Figure S2. Thermoresponsive properties of aqueous solutions of (a) PNIPAM and (b) PNIPAM-co-GMA monitored as changes of solution transparency with temperature.

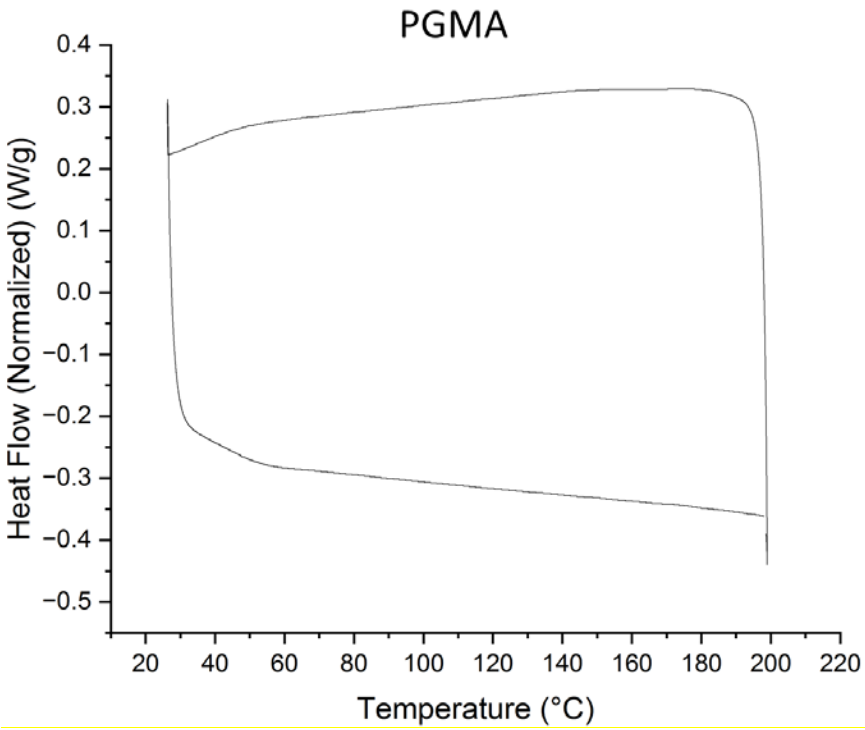

Figure S3. DSC analysis of PGMA

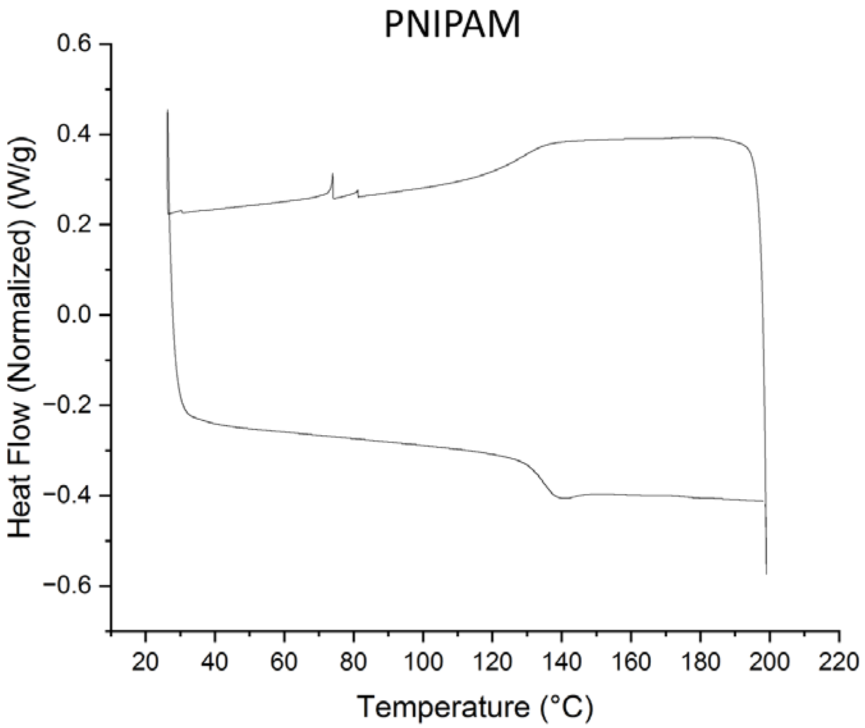

Figure S4. DSC analysis of PNIPAM

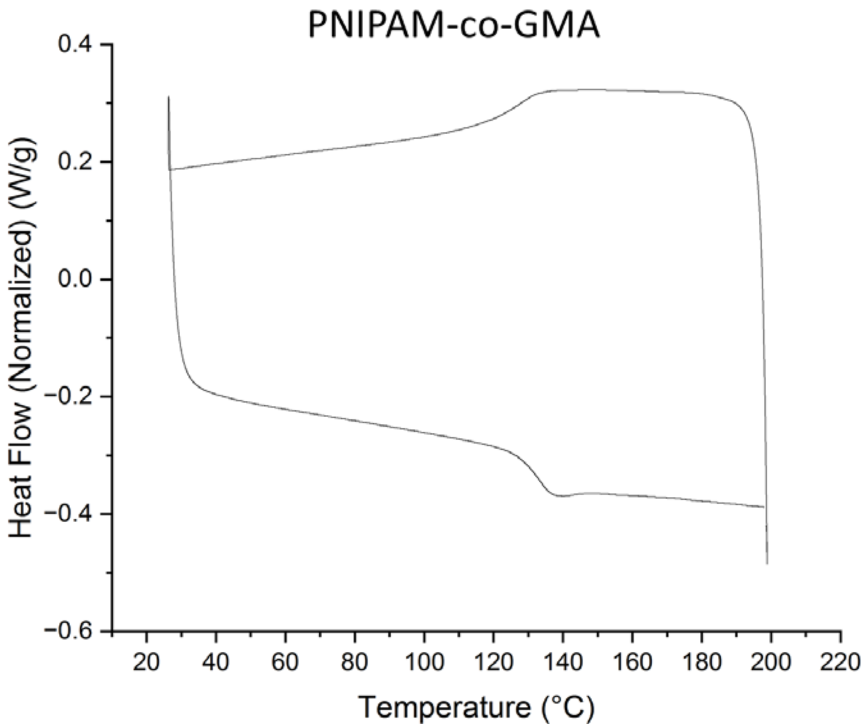

Figure S5. DSC analysis of PNIPAM-co-GMA

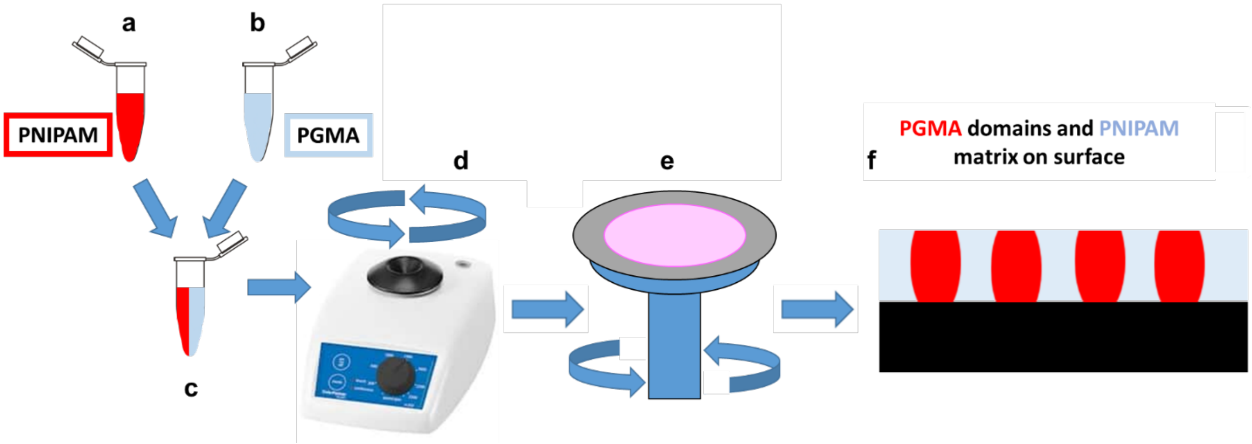

Figure S6. Step 1: Schematic presentation of the preparation of microstructured surfaces by spin-coating of (a) PNIPAM and (b) PGMA polymers in a (c) blend solution in dioxane. (d) Mixing the polymer blend and (e) Spin-coating of the mixture results in (f) phase separation and formation of the microstructured PGMA domains for further fabrication steps.

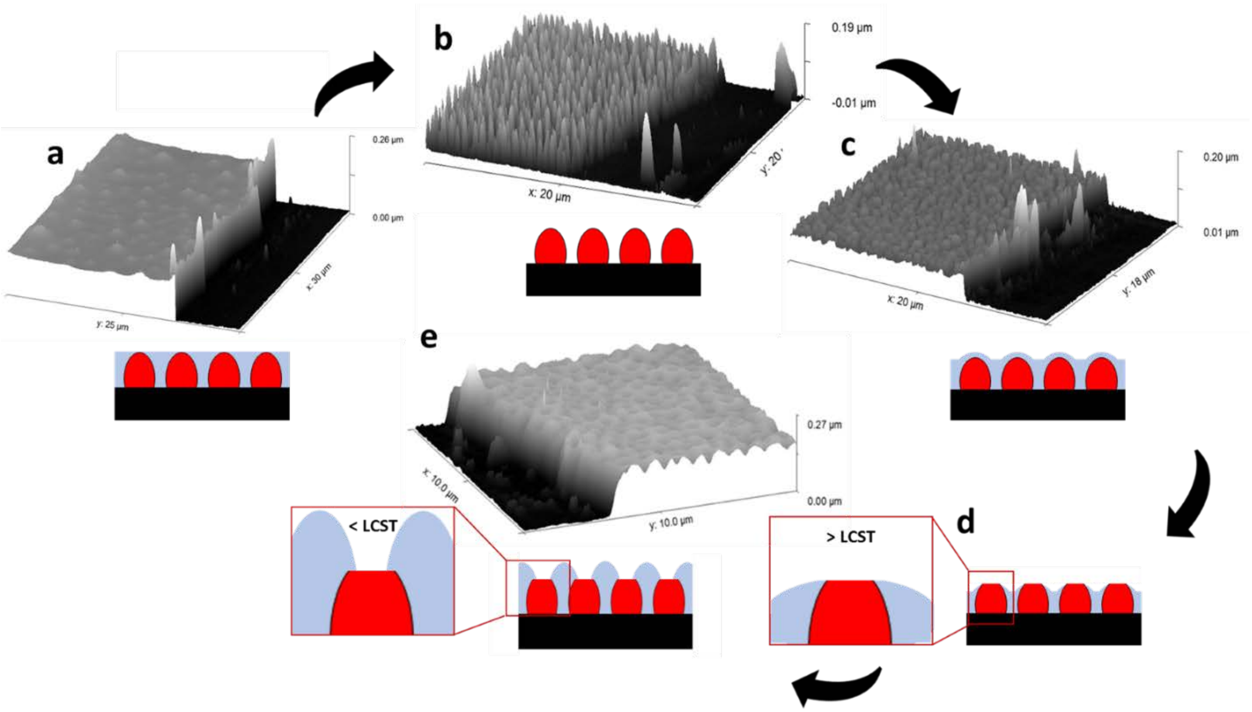

Figure S7. Step 2: Schematic presentation and SPM images of the (a) microstructured surface after Step 1; PGMA and PNIPAM are shown in red and blue colors, respectively; (b) microstructured surface made up of PGMA domains after washing off PNIPAM; (c) deposition PNIPAM-co-GMA (blue color) using spin-coating at room temperature; (d) etching of excess of PNIPAM-co-GMA to expose PGMA surface area for cell binding, (e) swelling of the PNIPAM-co-GMA matrix in water at T<LCST.

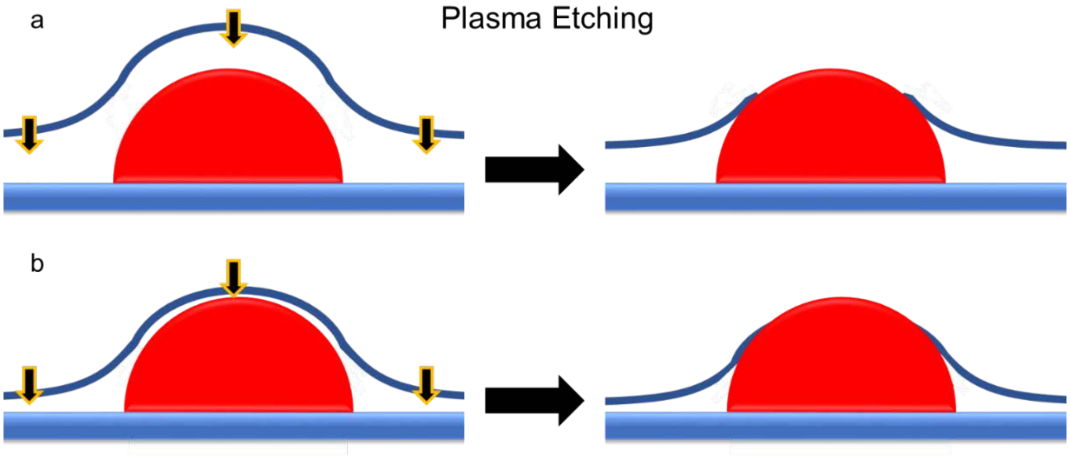

Figure S8. A schematic to explain the purpose of the plasma etching of the microstructured surface after deposition of PNIPAM-co-GMA over PGMA domains from solutions (a) 2 % and (b) 1 % to expose the PGMA surface to cells prior to testing.

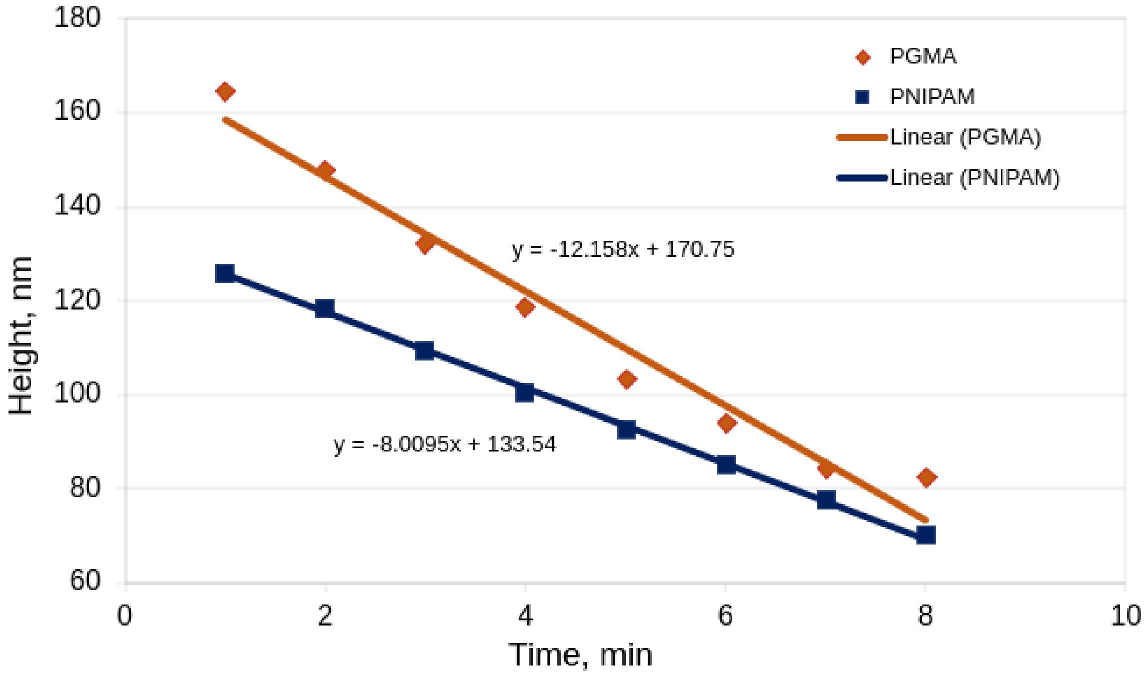

Figure S9. Plasma etching of PGMA and PNIPAM-co-GMA over time to establish the rate of PNIPAM-co-GMA matrix removal following PNIPAM-co-GMA deposition

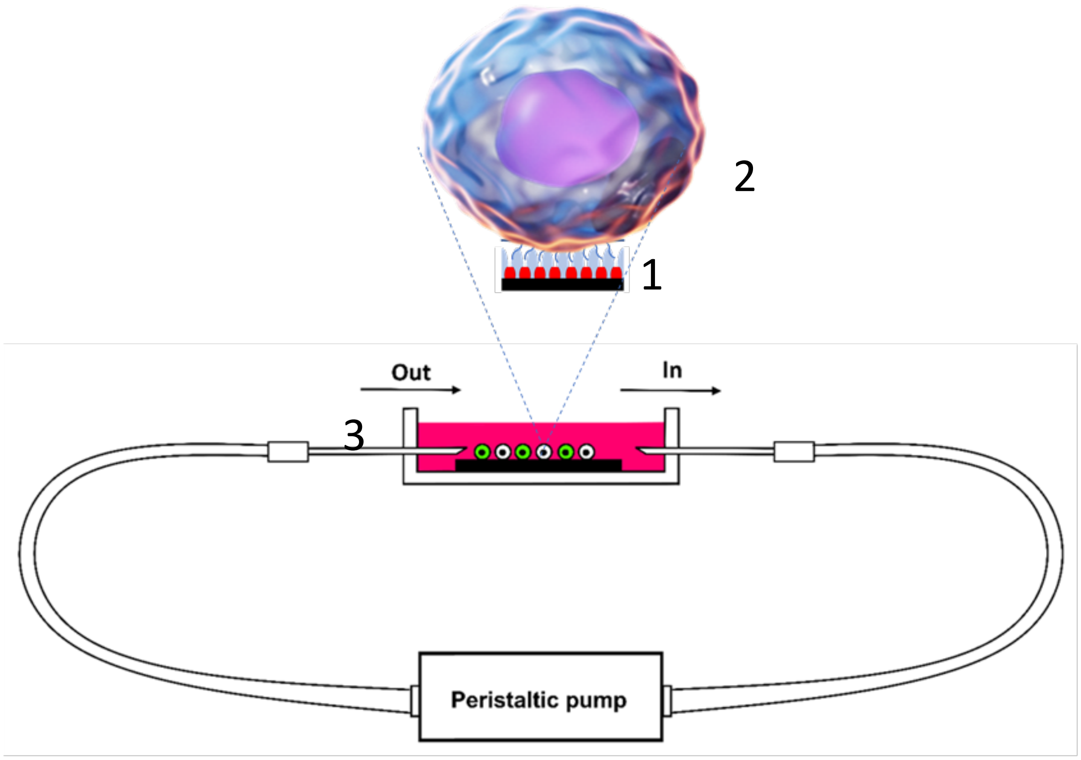

Figure S10. Schematic of the cell sorting setup: the sample (1) with the adhered cells (2) is mounted in the container with the cell media at T<LCST. The cell adhesion is weakened owing to the push-off force developed by PNIPAM-co-GMA swollen matrix. The media flow through a needle (3) is used to suspend loosely adhered cells.

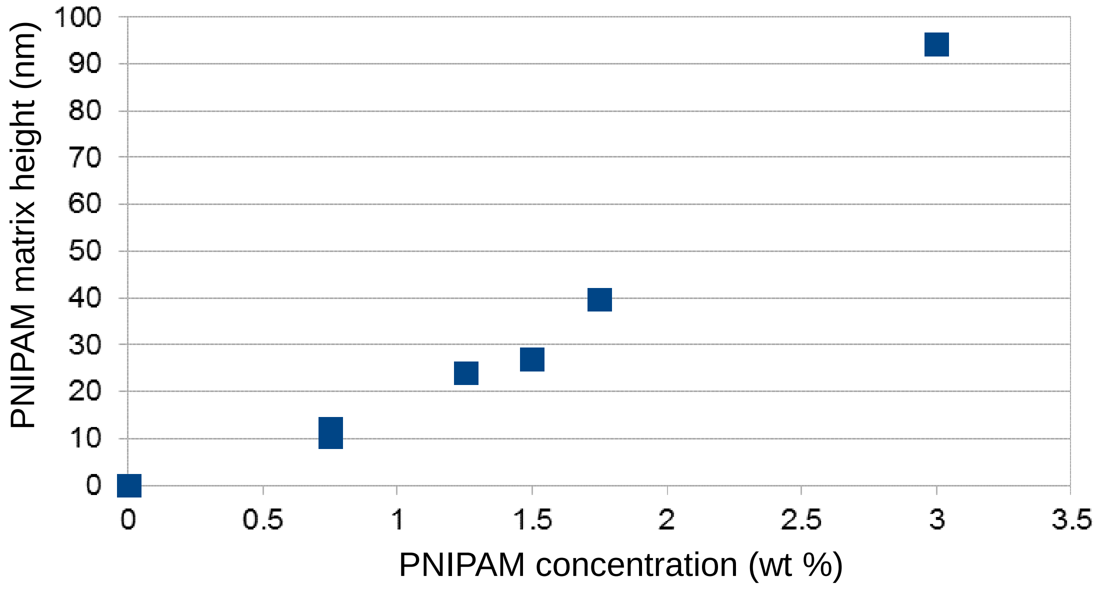

Figure S11. PNIPAM matrix height changes with an increase in the concentration of PNIPAM-co-GMA spin coating solution.

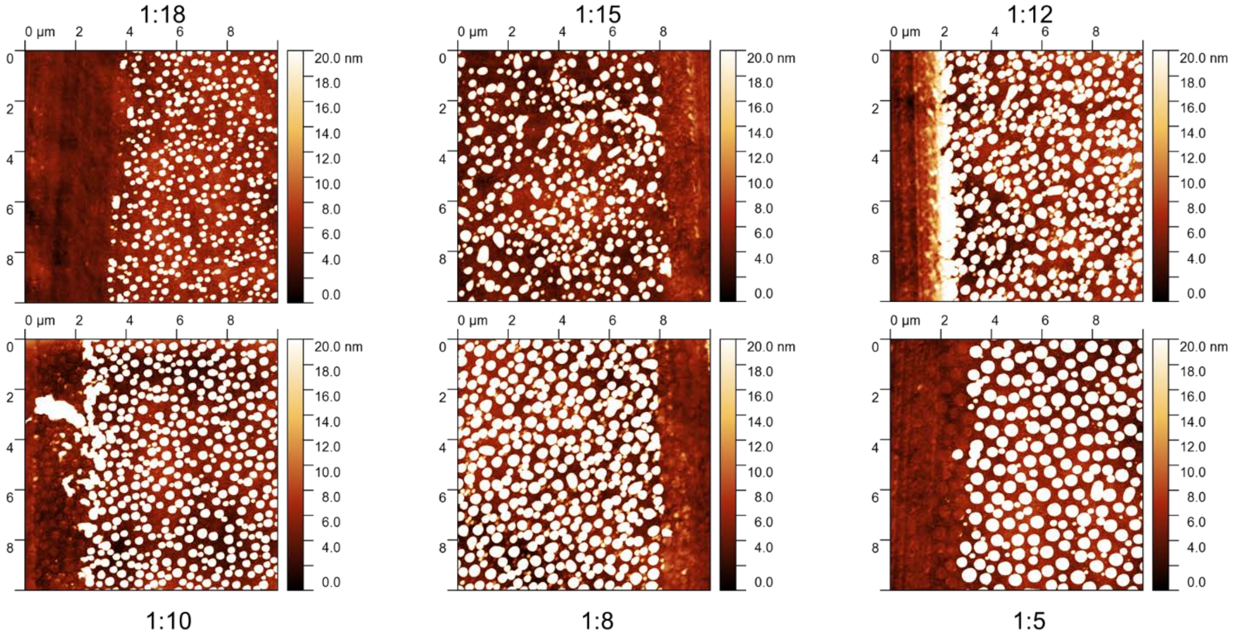

Figure S12. Representative SPM images of PGMA domains after Step 1 and washing out PNIPAM. The numbers represent the ratio between PNIPAM and PGMA solutions.

Table S1. Spherical cup geometry of the PGMA domain after Step 1: h_{slice} is threshold above the basal surface to mark individual domains, with only the part of the domain above the threshold used for analysis, R_d is the radius of the disc with the same projected area as the domain (or the radius of the equivalent circular section for each threshold height), R_{sp} is the curvature radius of the domain (or the radius of the imaginary sphere, calculated by spherical cap fitting of the domain), R_d' is the backward re-estimation of R_d, based on R_{sp} and H_{PGMA} and h_{slice}, assuming spherical cap approximation.

Experimental				Simulation			
h_{slice}, nm	R_d, nm	R_{sp}, nm	R_d', nm	h_{slice}, σ	R_d, σ	R_{sp}, σ	R_d', σ
f=0.065, H_{PGMA} = 52 nm				f=0.0625, H_{PGMA} = 7.76 σ (σ = ~7 nm)			
12	118.2	197.8	118.3	1	4.51	4.57	4.03
15	114	175.6	115.3	3	4.6	4.6	4.58
20	106.8	176.8	108.3	5	4.2	4.58	4.2
25	100.4	179.9	101.4				
30	91.9	184.0	93				
35	86.4	189.7	86.1				
40	79.5	198.7	76.3				
f=0.111, H_{PGMA} = 76 nm				f=0.0957, H_{PGMA} = 8.61 σ (σ = ~9 nm)			
12	160.3	240.6	161.2	1	4.89	5.38	5.06
15	160.1	205.7	158.3	3	5.67	5.67	5.49
20	153.3	205.9	153	5	5.11	5.42	5.16
25	146.4	207.2	146.9				
30	139.4	209.2	140.7				
35	133.6	212.1	134.1				
40	126.1	216.0	127.4				
f=0.200, H_{PGMA} = 104.5 nm				f=0.205, H_{PGMA} = 11.50 σ (σ = ~9 nm)			
12	211.7	292.4	210.5	1	7.94	8.25	8.45

15	208.3	243	209.3	3	9.03	9.05	8.65
20	206.6	244	206.6	5	8.45	8.74	8.38
25	201.7	246	202.8	7	7.51	8.52	7.59
30	197.4	247	197.7				
35	194.0	251	193.6				
40	190.5	253	190.9				

Table S2. Effect of 1 min of plasma treatment on the microstructured surface. H_{PGMA} is the median of the highest points of PGMA domain in air, with the inter-quartile range in parentheses; h_{PNIPAM} air is the height of PNIPAM layer in air, with the variance in parentheses.

Sample	Before plasma		After plasma		Etched height	
	H_{PGMA}, nm	h_{PNIPAM}, nm	H_{PGMA}, nm	h_{PNIPAM}, nm	ΔH_{PGMA}, nm	Δh_{PNIPAM}, nm
A1	69.9 (16.2)	27.6 (2.6)	57.1 (13.6)	16.7 (2.9)	12.8	10.9
A2	86.6 (6.6)	64.3 (2.6)	73.2 (6.3)	51.5 (2.3)	13.3	12.8
B1	45.2 (7.0)	29.8 (2.2)	41.5 (7.4)	19.4 (1.8)	3.6	10.4
B2	73.1 (4.5)	53.4 (2.3)	63.7 (7.0)	41.7 (1.8)	9.4	11.7
C1	37.7 (5.8)	21.8 (1.4)	32.6 (5.8)	16.0 (1.5)	5.1	5.8
C2	61.2 (5.0)	48.4 (4.4)	58.6 (4.1)	45.1 (1.4)	2.6	3.3

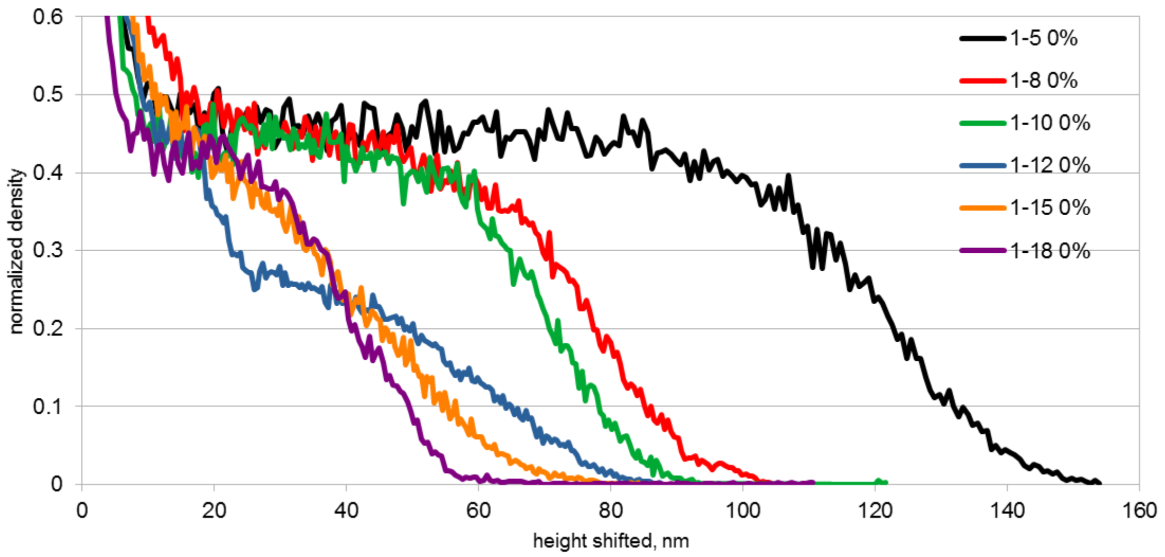

Figure S13. Height distribution (density) functions, normalized to demonstrate the distribution of PGMA domains, prepared from solutions with different PGMA:PNIPAM ratios.

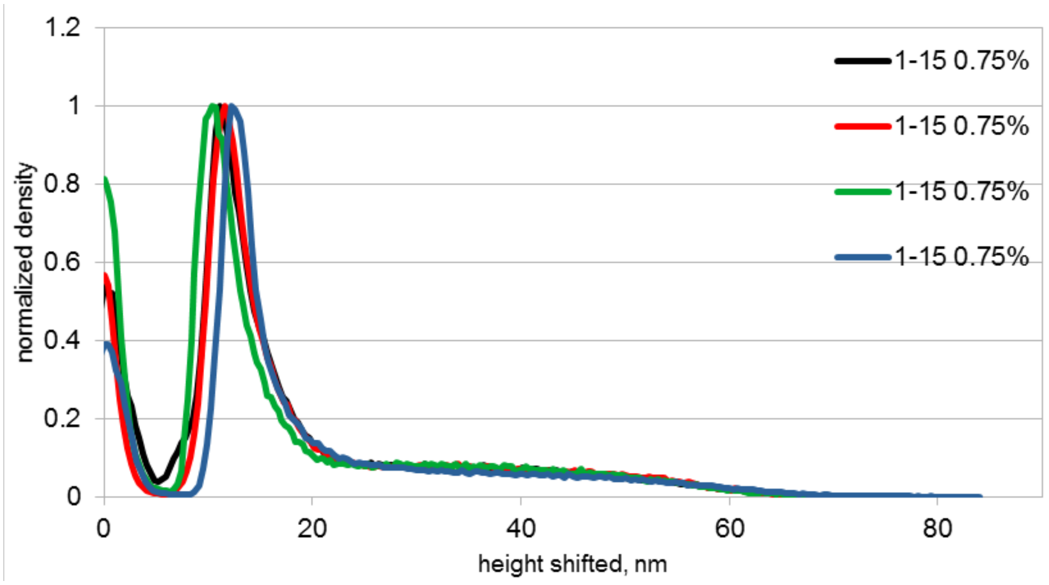

Figure S14. The height distribution of the PNPAM matrix on different locations of the same samples.

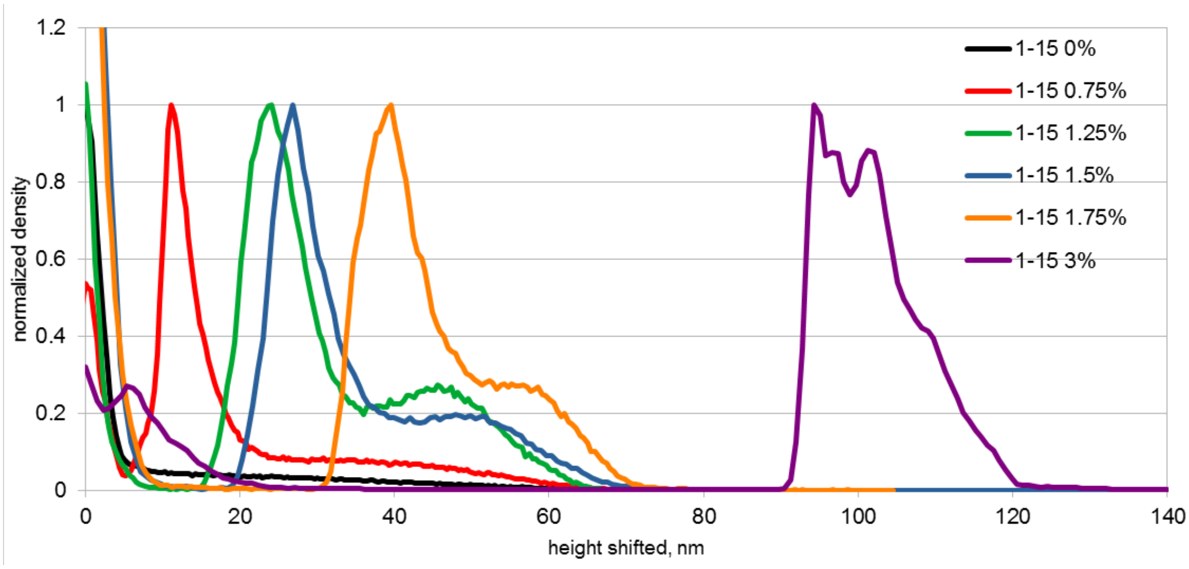

Figure S15. Height distribution functions of surfaces with 1:15 PGMA:PNIPAM ratio with different thicknesses of PNIPAM-co-GMA matrix and normalized to the same density of PNIPAM matrix in air. The first pick is associated with PNIPAM-co-GMA matrix, the shoulder is associated with a broad distribution of PGMA domains.

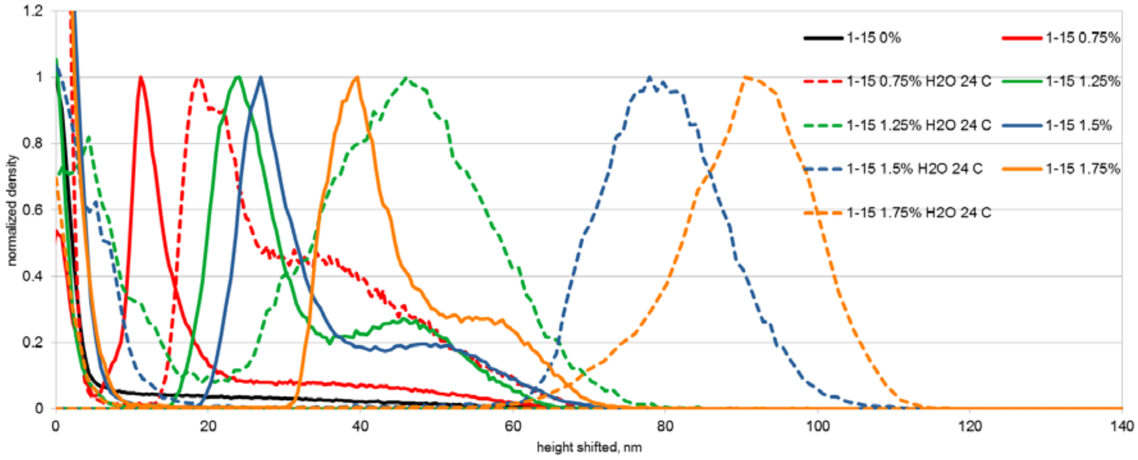

Figure S16. Height distribution functions of surfaces with 1:15 PGMA:PNIPAM ratio with different thicknesses of PNIPAM-co-GMA and normalized to the same density of PNIPAM matrix in air and in H_2O at 24 °C. The first pick is associated with PNIPAM-co-GMA matrix, the shoulder is associated with a broad distribution of PGMA domains.

Table S3. Cell Sorting by peristaltic pump results for flow ratios (Reference Cell:Detached Cell), the separation factor (SF), the number of cells before and after cell sorting, the final reference cell percentage of total cells, and the percentage change of the reference cell to the final percentage on microstructured surfaces at various incubation periods.

Cells mixtures	Sample	Incubation time	Ratio, >LCST (R1:1)	Ratio, <LCST (R2:1)	R1/R2 (SF)	Number of Cells Before	Number of Cells After	Reference Cell % After Sorting	Reference Cell % Change
RAW and 3T3	A1	20 min	5.9	23.2	3.9	2504	1886	95.9	10.4
		16 h	5.6	6.2	1.1	219	198	86.1	1.3
	A2	20 min	3.4	29.7	8.6	952	712	96.7	19.5
		16 h	5.3	5.5	1.1	284	239	84.6	0.5
	B1	20 min	6.1	22.3	3.7	936	804	95.7	9.8
		16 h	3.1	3.4	1.1	205	198	77.3	1.7
	B2	20 min	2.0	3.4	1.7	953	768	77.3	10.6
		16 h	5.7	6.5	1.2	207	176	86.7	1.6
	C1	20 min	1.8	4.4	2.6	460	322	81.5	17.2
		16 h	4.5	4.7	1.1	261	213	82.5	0.6
	C2	20 min	1.5	2.7	1.8	1300	864	73.0	13.0
		16 h	4.7	4.4	0.9	259	254	81.5	-1.0
RAW and HaCaT	A1	1 h	2.8	13.8	5	874	524	93.2	19.6
		16 h	4.5	4.9	1	432	380	83.1	1.2
	A2	1 h	3	34.8	11.6	174	95	97.2	22.2
		16 h	5.1	5.0	1	159	132	83.3	-0.3
	B1	1 h	4.1	32.2	7.8	476	310	97.0	16.6
		16 h	6.3	7.1	1.1	483	472	87.7	1.4
	B2	1 h	7.2	33.8	4.7	270	162	97.1	9.3
		16 h	4.8	5	1	285	261	83.3	0.6
	C1	1 h	3.2	175	54.7	376	176	99.4	23.2
		16 h	6.3	6.1	1	439	455	85.9	-0.4
	C2	1 h	2.3	47.6	20.7	218	98	97.9	28.2
		16 h	3.1	3.1	1	196	166	75.6	0.0
HaCaT and 3T3	A1	1 h	2.2	2.5	1.2	307	255	71.4	2.7
		16 h	1.6	1.8	1.2	203	213	64.3	2.7
	A2	1 h	2.2	2.8	1.3	425	295	73.7	4.9
		16 h	2.6	3.0	1.2	213	217	75.0	2.8
	B1	1 h	2.8	2.5	0.9	452	221	71.4	-2.3
		16 h	3.4	3.6	1.1	245	246	78.3	1.0
	B2	1 h	2.2	2.9	1.3	369	215	74.4	5.6
		16 h	2.4	2.4	1	209	214	70.6	0.0
	C1	1 h	1.5	1.6	1.1	413	263	61.5	1.5
		16 h	3.0	2.9	1	220	212	74.4	-0.6

	C2	1 h	2.5	42.2	16.9	764	15	97.7	26.3
		16 h	0	0	0	52	0	0.0	0.0

Details of the simulation models

Dissipative Particle Dynamics Model for the microstructured surface.

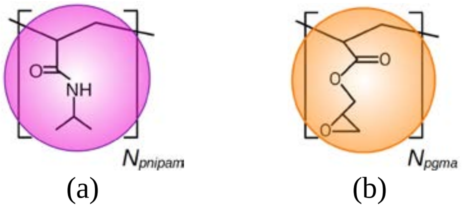

(a) (b)

Figure S17. Coarse-grained beads representing repeating units of PNIPAM (a) and PGMA (b).

The repeating unit of PGMA and PNIPAM chains are treated as a single soft bead of roughly 10 atoms each, as shown in Figure S17. The same is true for a group of water molecules. Therefore, a solvent is modeled explicitly. The diameters of both polymer and water beads, σ, their masses, m, the energy scale $k_B T$ (here k_B is the Boltzmann constant), the temperature, *T,* and the time unit, t, are all set as $\sigma = m = k_B T = t = 1$.[1] The magnitude of the repulsive force acting between the ith and jth beads is

$$F_{ij}^{C} = \begin{cases} a(1 - r_{ij}), & r_{ij} < 1 \\ 0, & r_{ij} \geq 1, \end{cases} \tag{1}$$

where a is the density and temperature-dependent repulsion strength and r_{ij} is the separation between the beads.

Besides conservative repulsive force, each pair of beads is subjected to the dissipative (friction), F_{ij}^{D}, and random, F_{ij}^{R}, forces with their respective magnitudes given by[2]

$$F_{ij}^{D} = -\gamma w^{D}(r_{ij})(\boldsymbol{r_{ij}} \cdot \boldsymbol{v_{ij}})\frac{1}{r_{ij}}, \qquad F_{ij}^{R} = \sigma w^{R}(r_{ij})\theta_{ij}\Delta t^{\frac{1}{2}}, \tag{2}$$

where $\boldsymbol{v_{ij}} = \boldsymbol{v_i} - \boldsymbol{v_j}$, $\boldsymbol{v_i}$ and $\boldsymbol{v_j}$ are respective velocities of the beads, and both magnitudes, $\sigma^2 = 2\gamma$, and the separation-dependent weight factors, $w^{D}(r_{ij}) = w^{R}(r_{ij})^2 = (1 - r_{ij})^2$, are interrelated[3]. $w^{D}(r_{ij}) = (1 - r_{ij})^2$ if $r_{ij} < 1$ and is zero otherwise, whereas θ_{ij} is the Gaussian distributed random variable. The integrity of a polymer chain is achieved via the harmonic bonding force

$$F_{ij}^{B} = -k_b(r_{ij} - b_{ij}), \tag{3}$$

With the choice of $k_b = 4$ and $b_{ij} = 0$.

For the polymer-polymer and water-water interaction, the parametrization by Groot and Warren[9] $a = 25$ is typically used. It is obtained for the bulk number density of beads equal to $\rho = \frac{N}{V} = 3$ by matching the model compressibility and that of water at normal conditions.[4] Polymers solubility and miscibility are tuned by an appropriate choice of the *a*, based on the Flory-Huggins mixing parameter χ[4, 5]. Its magnitude ranges from *a*=25 for ideally miscible beads (and good solubility, if one of the beads represents solvent), to *a*>35 for poorly miscible beads (poor solvent case), whereas the θ-point conditions are to be found at some intermediate value of *a** that is found below. Parametrization for the PNIPAM-water interaction is based on Ref. [2] and is covered in detail in our previous works[6] resulting in $a = 25.6$ for the swollen state at $T = 25°\mathrm{C} < \mathrm{LCST}$, and $a = 38$ for the collapsed state at $T = 37°\mathrm{C} > \mathrm{LCST}$. This model was validated before for both an isolated PNIPAM chain and a polymer brush[10]. Based on this work, the choice for the PNIPAM length, N_{pnipam} =100 beads, is made.

The choice for the PGMA length, N_{pgma}, is grounded on the requirement that the PGMA surface-grafted domains should be just higher than the PNIPAM matrix in its collapsed state, above LCST. We take into account that the PGMA chains are solidified in a dry state when a solvent eventually evaporates, terminating phase separation between PNIPAM and PGMA components. A suitable approximation of this condition in the realms of the DPD approach is the θ-solvent state. To find the magnitude *a** for the repulsive force between any monomer and a solvent that reproduces θ-solvent condition, we perform scaling analysis of the gyration radius, R_g~N^{ν}, of a single polymer chain in solvents of different quality. The results are shown in Figure S18, and we found that the scaling exponent ν=0.5, characteristic for θ-solvent, is achieved at *a**=27.5. This value agrees very well with the estimates provided by the analysis of the coil-to-globule transition undergoing by a PNIPAM chain[7], phase separation[11,12] and from the properties of heterostar polymers.[8]

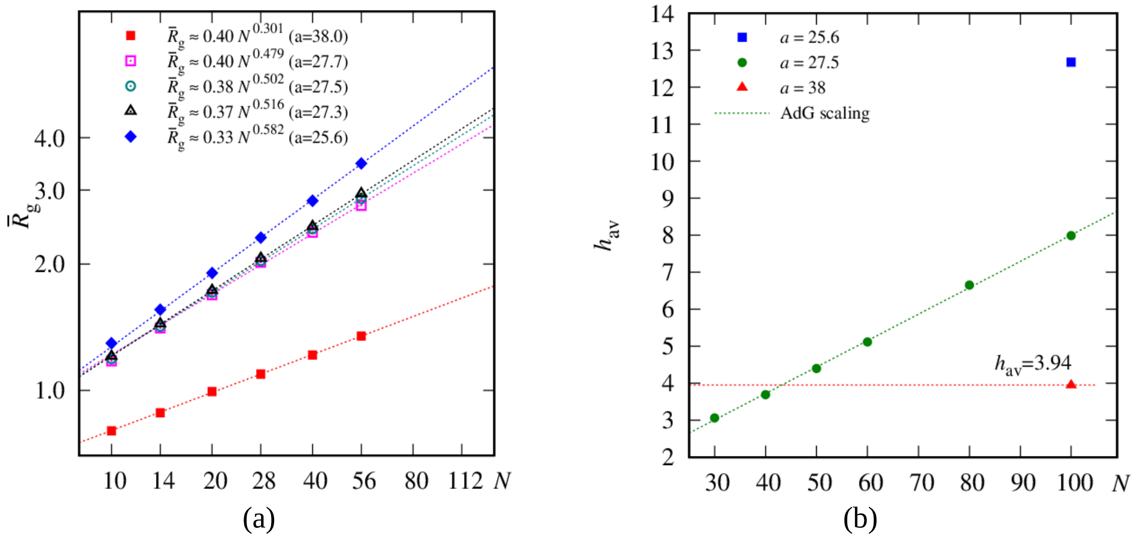

Figure S18. (a) Scaling law for the gyration radius, R_g~N^{ν}, of an isolated PNIPAM chain in solvents of various quality defined via the NIPAM-solvent repuslive strength, *a*. Here *N* denotes N_{pnipam}. The cases of a poor, *a*=38 (ν=1/3), and good, a=25.6 (ν~0.59), quality solvents are shown as well as the θ-solvent conditions (ν=0.5), which is found at *a*=27.5. (b) Average heights, h_{av}, for the PGMA grafted regions (green discs) at various chain lengths, N=N_{pgma}, at the θ-solvent condition. Average heights for the N_{pnipam}=100 grafted domains are indicated at both poor solvent (*a*=38, red triangle) and good solvent (*a*=25.6, blue square) conditions. Grafting density is ρ_g=0.2

everywhere. Green line indicates a fit to the Alexander - de Gennes scaling law; for more details see Ref. (10).

An average height, h_{av} of the PGMA surface-grafted domains in θ-solvent (a=a*=27.5) at various chain lengths, N_{pgma}, is presented in Figure S18b with green solid circles. It is compared against its counterpart for the PNIPAM of the length N_{pnipam}=100 at poor solvent (h_{av}=3.94 at a=38, red triangle) and good solvent (h_{av} = 12.7 at a = 25.6, blue square) conditions, all at grafting density of ρ_g = 0.2. Green dotted line shows the Alexander - de Gennes scaling law for grafted polymer which is found to provide an excellent fit to obtained data. The green dotted line crosses the line h_{av} = 3.94 at about N_{pgma} = 43, hence a safe choice for N_{pgma}=60 is made in this study.

Initially we used the model to analyze the formation of the microstructure surfaces due to the phase separation between PGMA and PNIPAM. Such phase separation is initiated by strong repulsion, a_{sep}>35, between the monomers of these two species. Simulations are performed within the simulation box of dimensions 80x80x30 in DPD length units (set by the diameter of equally-sized soft beads). We examined a wide interval of values, from a_{sep}=38 to 80, finding only minor differences in the separation dynamics. Therefore, the value a_{sep}=60 is used hereafter. Both polymers are in the condition of the θ-solvent (a=a*=27.5). Phase separation is undertaken during $500{\cdot}10^3$ DPD steps and then terminated, mimicking evaporation of a solvent in an implicit way.

Mapping simulation data onto the experimental ones, one can find scaling relations between both. This is done in Figure S19. Firstly, we found the length scaling factor of $\sigma \approx$ 9nm, to match the $<H_{pgma}>$ data.

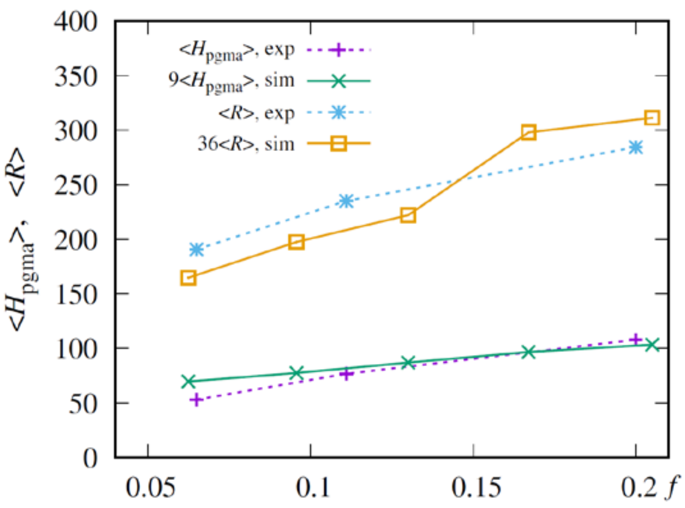

Figure S19. Matching simulation data for the average domain height, $<H_{pgma}>$, and average sphere radius, $<R>= R_{sp}$, (both denoted “sim” next to them) with their experimental counterparts (both denoted via “exp”).

The effect of pinning of PNIPAM on the solid domain walls. The PNIPAM-co-GMA was pinned randomly with fixed densities ρ_{ps} and ρ_{pd}, and allowed to equilibrate for 200000 steps at the θ-solvent condition (a=27.5). Then with probability p=10^{-6} active beads of PNIPAM-co-GMA which

are close enough to each other are crosslinked (a bond is formed between them). The fraction of active beads is set to be 30% initially, and those uncrosslinked active beads can be turned into simple PNIPAM beads if crosslinking stopped. The fraction of the crosslinked beads is $v_{cr}= 2N_b/N_{pnipam}100\%$, where N_{pnipam} is the number of all beads, including crosslinked. The values of v_{cr} considered are set to be v_{cr}~7% and v_{cr}~14%, with error less than 1%. The PNIPAM-co-GMA prepared in such a way was used for simulations in good (a=25.6) and poor (a=38) solvent condition, mimicking swelling of PNIPAM-co-GMA matrix at (T<LCST) and collapsing at T>LCST case.

Using the results of the simulation, where the pinning density on domains is the most important effect, one can develop speculative theoretical description of swelling ratio dependence on domains area fraction. Let us assume that there are two different average heights of PNIPAM-co-GMA gel, one at the substrate, h, and another on the domains, H. This can be due to different pinning densities ρ_g=0.2 and ρ_{pd}=0.6 on them, and additionally due to the height of domains themselves. Then, the swelling ratio as a function of fraction of the area covered by PNIPAM on domains, σ, would be as follows: $h_1/h_2(\sigma) = (h_1\rho_{ps}(1-\sigma)+\sigma\rho_{pd}H_1)/(h_2\rho_{ps}(1-\sigma)+\sigma\rho_{pd}H_2)$, where indices 1,2 correspond to conditions below LCST and above it, respectively. An effect of the height of domains themselves can be accounted for by introducing coefficients c_1 and c_2 as follows $h_i = c_i h_{i.pln}$. The meaning of coefficients, c_i, is of how much the average height on domains increased compared to the same layer if it would be on the plane. The reasonable range for the coefficient c_2 is $2<c_2<3$, as the height of collapsed PNIPAM is expected to be equal or smaller than the domain height. Similarly, one can choose c_1 in $1<c_1<2$, as swollen gel is expected to be equal or higher than the domains. Finally, let us assume that scaling $h\sim\rho^n$, similar to the Alexander-de Gennes law, takes place for weakly crosslinked and pinned gel. The exponent $h\sim\rho^n$ can be inferred from the simulations of uniform pinned gel layer, and for v_{cr}~6% was found to be n=0.6 in good solvent, and n=0.9 in bad one, see Figure S20(a). Then, one arrives at the expression for swelling ratio $h_1/h_2(\sigma)= c_0(0.2^{1.6}(1-\sigma)+\sigma c_1 0.6^{1.6})/(0.2^{1.9}(1-\sigma)+\sigma c_2 0.6^{1.9})$, where ρ_{pd}=0.6 and ρ_{ps}=0.2. The coefficient c_0 is harder to estimate and it is not very important for our analysis. Here we chose c_0=3. In Figure S20(b) there are shown the dependence of swelling ratio on the area fraction of PNIPAM covered domains, σ, for various values of the parameters c_1 and c_2.

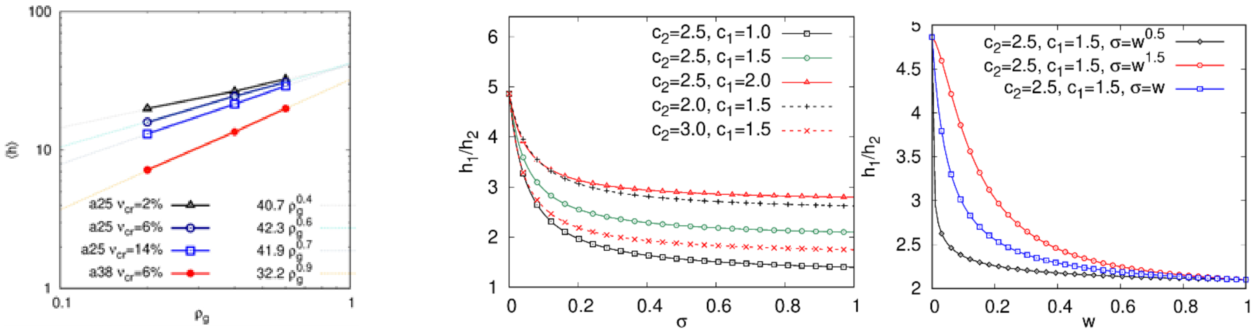

(a) (b) (c)

Figure S20. The Alexander-de Gennes-like law inferred from simulations (a) of gel formed from a crosslinked brush. The swelling ratio as a function of area fraction of PNIPAM covered domains, σ, (b), is shown for different values of parameters c_1 and c_2. The swelling ratio as a function of weight fraction of PGMA in solution, w, assuming different power-like dependencies of σ on w, is shown in (c).

As can be seen from both Figure S20(b) and (c), qualitatively, the pinning effect leads to a decrease of swelling ratio with an increase of domains covered with PNIPAM area, σ, (b), or weight fraction of PGMA, w, as in (c). This behavior is observed for the range of parameters c_1 and c_2 considered (b), and assuming different dependencies of area fraction σ on w (c).

Monte Carlo simulations of cell sorting

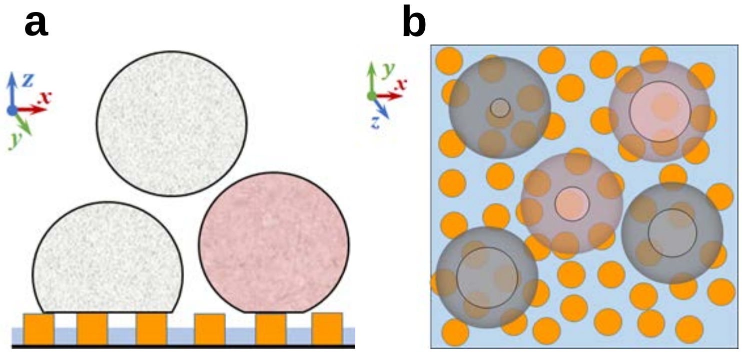

Figure S21. Schematic representation of the model of cells at a micropstructured surface: (a) Side view, and (b) top view of the model system. Orange squares or circles indicate adhesive PGMA domains, the blue areas represent PNIPAM-co-GMA matrix; gray and pink circles correspond to two types of cells; the outer circles depict the cell contours, while the inner circles show the contact area between the cells and the surface. Transparency is used to make visible the adhesive domains located beneath the cells.

The potential energy of a cell located in the position with coordinates $(x_i, \mathrm{y}_{i}, z_i)$ is described by the potential of interaction consisting of three contributions:

$$U_\alpha(x_i, \mathrm{y}_{i}, z_i) = \begin{cases} U_\alpha^{att}(x_i, \mathrm{y}_{i}, z_i) + U_\alpha^{rep}(x_i, \mathrm{y}_{i}, z_i) + U_\alpha^{gra}(z_i), & z > z_{\min} \\ \infty, & z < z_{\min} \text{ or } z > L_z \end{cases} \quad (4)$$

where $U_\alpha^{att}(x_i, \mathrm{y}_{i}, z_i)$ results from the attractive interaction between a cell and domains, $U_\alpha^{rep}(x_i, \mathrm{y}_{i}, z_i)$ describes the repulsion due interaction of a cell with the swollen polymer phase, and $U_\alpha^{gra}(z_i)$ takes into account the gravity.

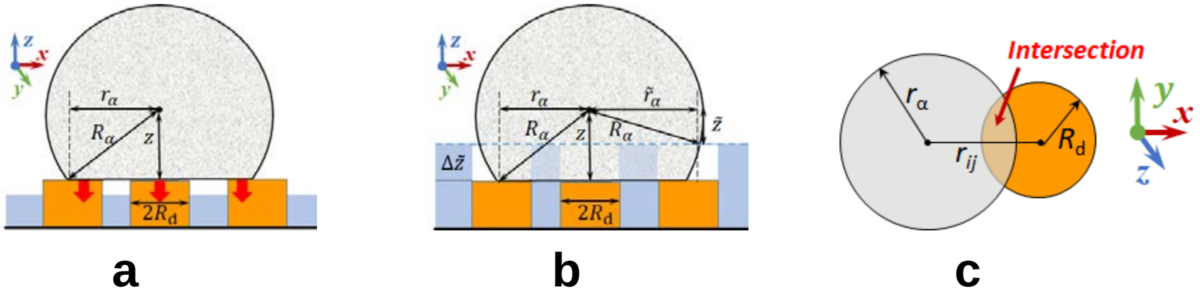

Figure S22. Schematic representation of the model of cell adhesion and detachment on a micropatterned surface. Orange squares or circles denote cylindrical adhesive domains, the blue area represents the polymer phase, and gray circles depict a cell. (a) A cell attached to the adhesive domains when the polymer phase is in the collapsed state. (b) A cell on the surface when the polymer phase is in the swollen state. Red arrows indicate the direction of attractive interaction to the attractive domains. Dark blue arrows show the direction of repulsive interaction from the surface due to the swollen polymer phase. (c) Intersection of two circles depict the area of the cell (gray) in contact with the surface and the top surface of the adhesive domain (orange).

The attractive interaction can be calculated from a sum of all contacting surface areas of a cell with neighboring domains $s_{\alpha d}(r_{ij})$ multiplied by the adhesion parameter $A_{\alpha d}$, which is specific for each type of cells:

$$U_\alpha^{att}(x_i, \mathrm{y}_i, z_i) = \sum_{\{j|r_{ij} \leq r_{\alpha d}\}} A_{\alpha d} s_{\alpha d}(r_{ij}), \tag{5}$$

where $\alpha = 1 \text{ or } 2$ – sort of cell, and 'd' denotes domain. The distance between centers of cell i and a domain j in XY-plane is determined as $r_{ij} = \sqrt{(x_i - x_j)^2 + (y_i - y_j)^2}$. The maximum distance r_{ij} is limited to $r_{\alpha d} = r_\alpha + R_d$, where R_d is the radius of a domain and $r_\alpha = \sqrt{R_\alpha^2 - z_i^2}$ is the radius of circular intersection of the cell sphere and patterned surface. This intersection takes place at $z = 0$, when a cell center is located at the distances to the surface $z_i < R_\alpha$, where R_α is the radius of cell. It is worth noting that the distance z_i cannot be smaller than $z_{\min}$, which also limits the deformation extent of a cell. There two extreme cases exist, when $z_{\min} = R_\alpha$ and no deformation is allowed, and when $z_{\min} = 0$ a half of a cell can deform turning itself into a hemisphere. Below, we will consider some intermediate values of $z_{\min}$ corresponding to a partial deformation.

The contacting surface area of a cell i and a neighboring domain j can be calculated form the formula derived for the intersection area of two circles with radii r_α and R_d at the distance r_{ij}:

$$s_{\alpha d}(r_{ij}) = \begin{cases} \min(s_\alpha, s_d), & r_{ij} \leq r_\alpha - R_d \\ s_{\text{int}}(r_{ij}), & r_\alpha - R_d < r_{ij} \leq r_\alpha + R_d, \\ 0, & r_{ij} > r_\alpha + R_d \end{cases} \tag{6}$$

where

$$\mathrm{s}_{\text{int}}(r_{ij}) = R_\alpha^2 \arccos\left(\frac{r_{ij}^2 + r_\alpha^2 - R_d^2}{2 r_\alpha r_{ij}}\right) + R_d^2 \arccos\left(\frac{r_{ij}^2 + R_d^2 - r_\alpha^2}{2 R_d r_{ij}}\right) - \frac{1}{2}\sqrt{[(r_\alpha + R_d)^2 - r_{ij}^2][r_{ij}^2 - (R_d - r_\alpha)^2]}, \tag{7}$$

and $s_\alpha = \pi r_\alpha^2$ – the area of circular interface between the cell sphere and patterned surface, $s_d = \pi R_d^2$ – the area of the top surface of a domain.

The repulsive contribution to the potential energy of a cell $U_\alpha^{rep}(x_i, \mathrm{y}_i, z_i)$ can be calculated in a similar manner as of an attractive one, but it is a bit more complex and should contain two terms:

$$U_\alpha^{rep}(x_i, \mathrm{y}_i, z_i) = B_{\alpha p} \tilde{s}_\alpha - \sum_{\{j|r_{ij} \leq r_{\alpha d}\}} B_{\alpha p} \tilde{s}_{\alpha d}(r_{ij}), \tag{8}$$

where p indicates 'polymer' and $B_{\alpha p}$ is the parameter of repulsive interaction of a cell with the polymer phase. The first term in (8) produces a uniform repulsive field towards a cell and depends only on the area of equatorial cross section of a cell i, while the second term excludes regions, which are not accessible for the polymer phase due to a presence of domains beneath the cell. As is seen the second term in (8) recalls the expression (2), but with the different $\tilde{s}_{\alpha d}(r_{ij})$, since it depends on the distance of cell' center to the polymer surfaces $\tilde{z}_i$. On the other hand, $\tilde{s}_{\alpha d}(r_{ij})$ is calculated using the expressions to (7) (8), but for different $\tilde{r}_{\alpha d} = \tilde{r}_{\alpha} + R_d$ and $\tilde{s}_{\alpha} = \pi\tilde{r}_{\alpha}^2$, where $\tilde{r}_{\alpha} = \sqrt{R_{\alpha}^2 - \tilde{z}_i^2}$. To distinguish variables, we mark them with tilde for the calculation of interaction between a cell and polymer phase, and without tilde in the case of cell-domain interaction.

The last contribution to the potential energy in (1), $U_{\alpha}^{gra}(z_i)$, depends only on z_i-coordinate of a cell i as a linear function and describes the gravity field driving a cell towards the patterned surface:

$$U_{\alpha}^{gra}(z_i) = G_{\alpha} z_i. \qquad (9)$$

where G_{α} – the gravity energy parameter.

The proposed model is realized through Monte Carlo simulations using the common Metropolis algorithm[13]. The system of cell particles is considered in a simulation box with the dimensions $L_x \times L_y \times L_z$ and the periodic boundary conditions applied along X and Y axes, while along Z-axis the system is bounded by parallel hard walls at $z = 0$ and $z = L_z$. The dimensions of simulation box are chosen equal to $L = L_x = L_y = 240.0$ and $L_z = 240.0$. Here and below, all sizes and distances in our study are presented in reduced units. In case of necessity they can be scaled into real units by assuming that a typical size of cell in this units is equal to 10.0.

The micropatterned surface is constructed as array of parallel spherocylinders oriented orthogonally to the surface and their centers are distributed randomly along XY-plane at the bottom of simulation cell. No overlap between spherocylinders is allowed and their positions remain unchanged during a simulation. We set the same diameter $D_d = 2R_d$ for all domains, and consider it in the range of $D_d = 0.5 - 2.0$. We also introduce the coverage fraction of surface domains $\sigma_d = \pi D_d^2 \rho_d / 4$, where $\rho_d = N_d / L^2$ – the surface density of domain and N_d – the number of domains at the surface. To investigate the effect of domain size the fraction of surface domains fixed at $\sigma_d = 0.48$ for the different D_d.

The cells are represented as a two-component mixture of hard-sphere particles, and no more other specific interactions between them are taken into account at this level of modeling. The interaction of cells with patterned surface is calculated according to the scheme described in the previous subsection. We consider two types of cells $\alpha = 1$ and 2, which differ only by the strength of attraction with the domains, i.e. by the adhesion parameter $A_{\alpha d}$, where $A_{1d} = -0.4$ and $A_{2d} = -0.3$. The energetic quantities are also used in the reduced units.

We assume that the cells have an equal size, i.e. $D_1 = D_2 = 10.0$. On the other hand, the repulsion parameter $B_{\alpha p}$ is taken the same for both types, since it depends rather on the elastic properties of polymer phase than on specific interactions between the polymer chains and the cell membrane. We consider the parameter $B_{\alpha p}$ in the range $B_p = B_{1p} = B_{2p} = 0.0 - 0.3$, in which the detachment of the cells can be expected.

The gravity parameter G_α present in (9) is also taken the same for both types of cells $G_\alpha = 0.0025$, and it is set rather small compared to the surface-cell interactions.

The model parameters used in the simulations are collected in Table S4.

All snapshots (Figure S23-Figure S27) and SI Videos obtained from our simulations are created with the help of OVITO software[14].

Table S4. Model parameters used in simulations.

D_d	σ_d	D_1	D_2	A_{1d}	A_{2d}	z_{min}	$\Delta\tilde{z}$	G_α	B_p
0.5-2.0	0.48	10.0	10.0	-0.4	-0.3	2.5	0.1	0.025	0.00-0.30

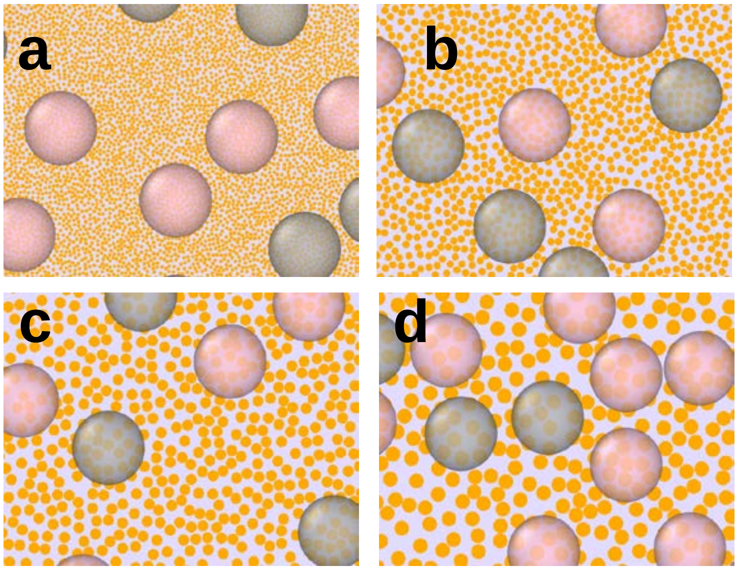

Figure S23. Cells (gray and pink spheres) on the microstructured surface with randomly distributed adhesive PGMA domains (orange circles) of different sizes $D_d = (a)0.5$, (b) 1.0, (c) 1.5, and (d) 2.0.

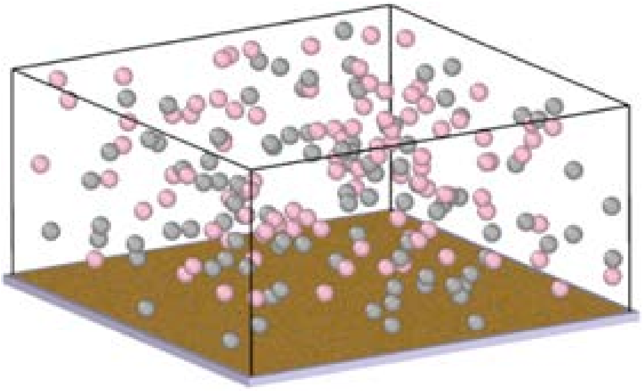

Figure S24. Cells (gray and pink spheres) at the initial step of the first stage before adsorption started ($D_d = 1.0$).

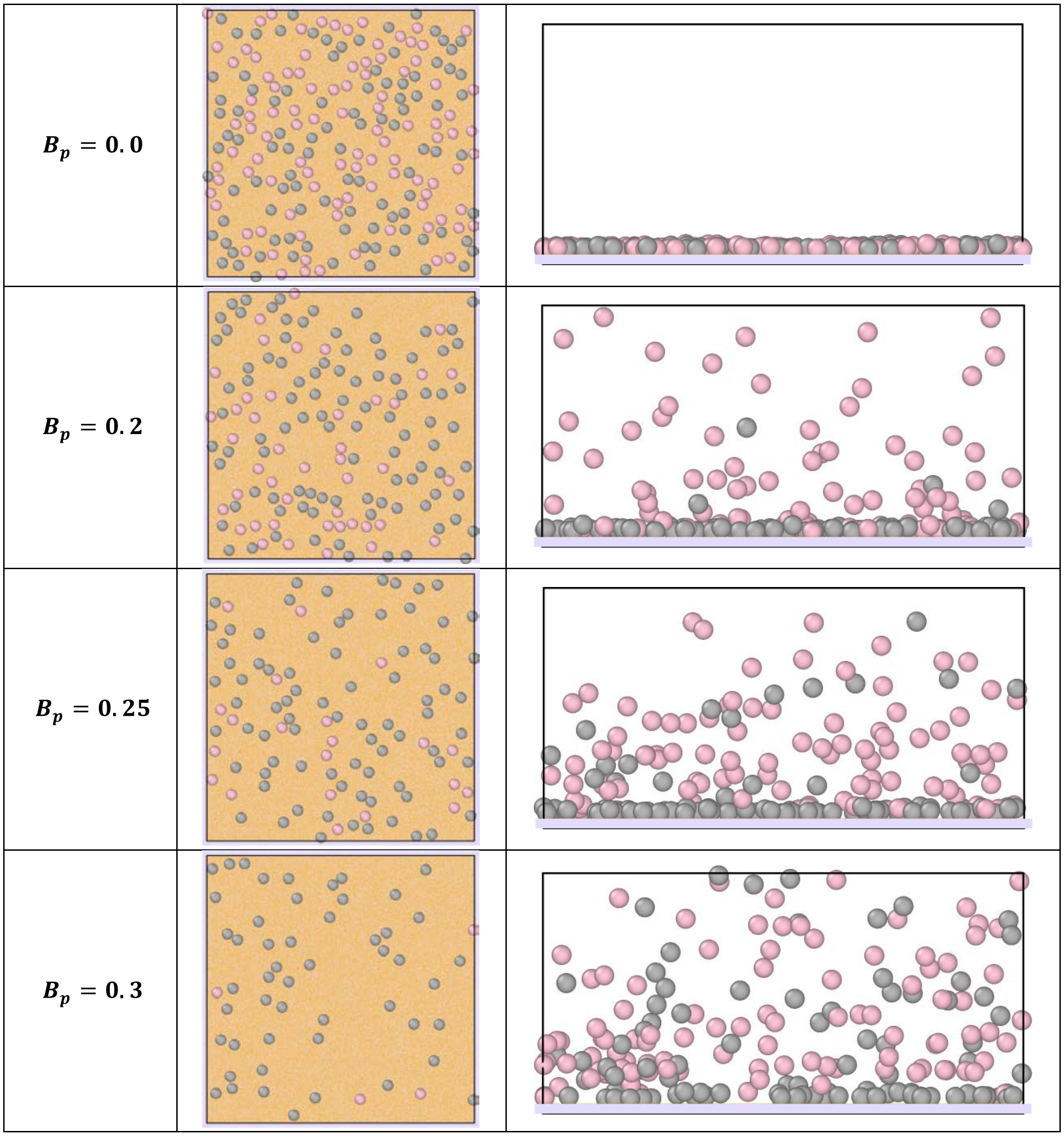

Figure S25. Snapshots of simulation cells for the different repulsive parameters $B_p = 0.0$, 0.2, 0.25 and 0.3 after 2M steps, when the domain size is set to $D_d = 0.5$.

$B_p = 0.0$	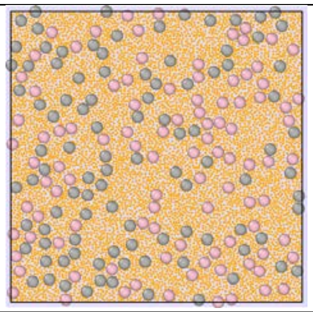	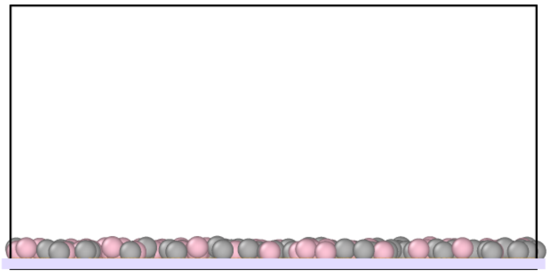
$B_p = 0.2$	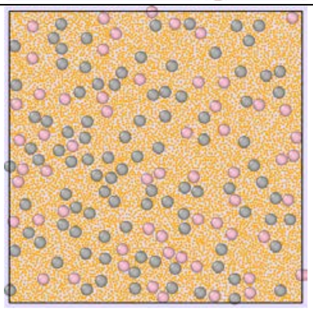	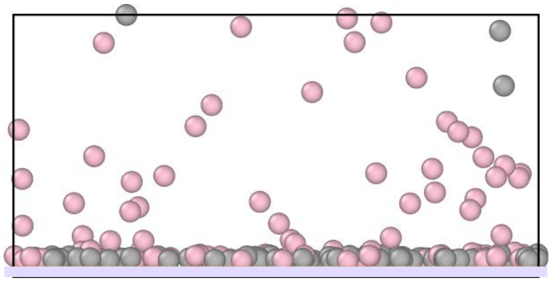
$B_p = 0.25$	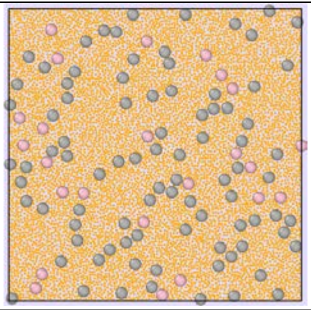	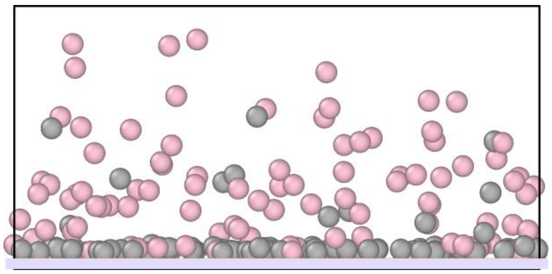
$B_p = 0.3$	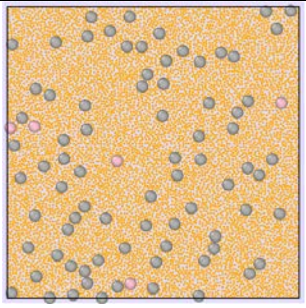	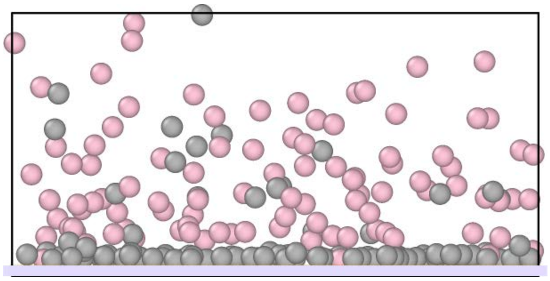

Figure S26. Snapshots of simulation cells for the different repulsive parameters $B_p = 0.0$, 0.2, 0.25 and 0.3 after 2M steps, when the domain size is set to $D_d = 0.5$.

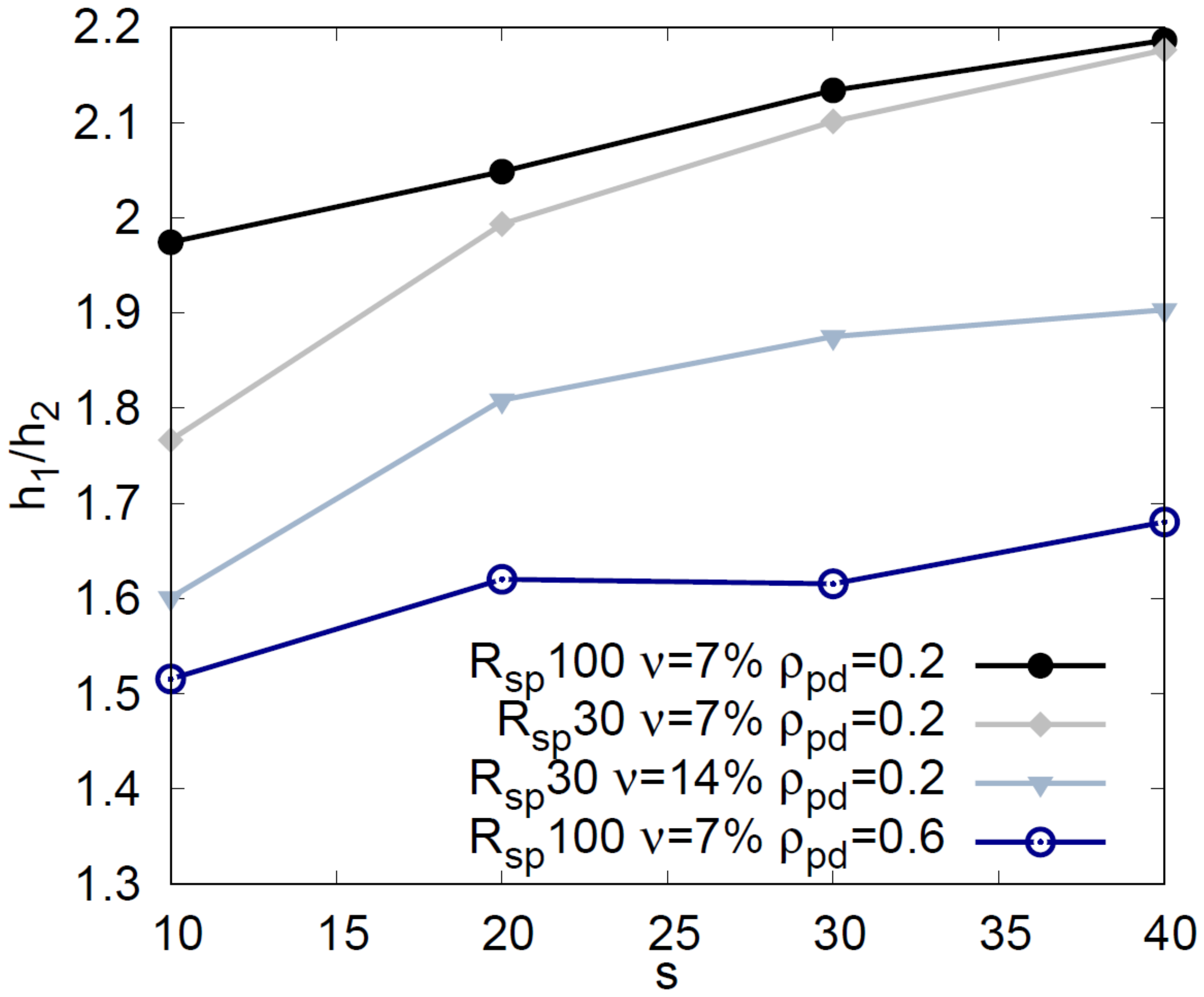

Figure S27. The swelling ratio depends on various parameters, as in Figure 6, but here, for an equilibrated network, the trends are the same, and similarly, the effect of grafting density is most significant.

REFERENCES

(1) Groot, R. D.; Warren, P. B. Dissipative particle dynamics: Bridging the gap between atomistic and mesoscopic simulation. *J. Chem. Phys.* **1997**, *107* (11), 4423-4435. DOI: 10.1063/1.474784 acccessed 12/20/2023).

(2) Soto-Figueroa, C.; Rodríguez-Hidalgo, M. d. R.; Vicente, L. Dissipative particle dynamics simulation of the micellization–demicellization process and micellar shuttle of a diblock copolymer in a biphasic system (water/ionic-liquid). *Soft Matter* **2012**, *8* (6), 1871-1877, 10.1039/C1SM07037A. DOI: 10.1039/C1SM07037A.

(3) Español, P.; Warren, P. Statistical Mechanics of Dissipative Particle Dynamics. *Europhys. Lett.* **1995**, *30* (4), 191. DOI: 10.1209/0295-5075/30/4/001.

(4) Groot, R. D.; Warren, P. B. Dissipative particle dynamics: Bridging the gap between atomistic and mesoscopic simulation. *The Journal of Chemical Physics* **1997**, *107* (11), 4423-4435. DOI: 10.1063/1.474784 (acccessed 4/27/2025).

(5) Groot, R. D.; Madden, T. J. Dynamic simulation of diblock copolymer microphase separation. *The Journal of Chemical Physics* **1998**, *108* (20), 8713-8724. DOI: 10.1063/1.476300 (acccessed 4/27/2025).

(6) Badenhorst, R.; Makaev, S.; Yaremchuk, D.; Sajjan, Y.; Sulimov, A.; Reukov, V. V.; Lavrik, N. V.; Ilnytskyi, J.; Minko, S. Reversible Binding Interfaces Made of Microstructured Polymer Brushes. *Langmuir* **2024**, *40* (13), 7008-7020. DOI: 10.1021/acs.langmuir.4c00062.

(7) Guo, J.; Liang, H.; Wang, Z.-G. Coil-to-globule transition by dissipative particle dynamics simulation. *The Journal of Chemical Physics* **2011**, *134* (24). DOI: 10.1063/1.3604812 (acccessed 4/27/2025).

(8) Kalyuzhnyi, O.; Ilnytskyi, J. M.; Holovatch, Y.; von Ferber, C. Universal shape characteristics for the mesoscopic star-shaped polymer via dissipative particle dynamics simulations. *Journal of Physics: Condensed Matter* **2018**, *30* (21), 215101. DOI: 10.1088/1361-648X/aabc16.

(9) Groot R. D.; Warren P. B. Dissipative particle dynamics: Bridging the gap between atomistic and mesoscopic simulation. *The Journal of Chemical Physics* **1997**, 107 (11): 4423–4435. DOI: 10.1063/1.474784

(10) Yaremchuk D.; Kalyuzhnyi, O.; Ilnytskyi, J. Modelling thermoresponsive polymer brush by mesoscale computer simulations Modelling. *Condensed Matter Physics* **2023**, 26 (3), 33302. DOI: 10.5488/CMP.26.33302

(11) Buhot A.; Krauth W. Phase separation in two-dimensional additive mixtures. *Physical Review E* **1999**, 59(3), 2939-2941. DOI: 10.1103/PhysRevE.59.2939

(12) Harinadha Gidituri, D. Vijay Anand, Srikanth Vedantam, Mahesh V. Panchagnula. Dissipative particle dynamics study of phase separation in binary fluid mixtures in periodic and confined domains. *The Journal of Chemical Physics* **2017**, 147 (7): 074703. DOI: 10.1063/1.4999096

(13) Allen, M. P.; Tildesley, D. J. Computer simulation of liquids. Oxford university press, **2017**.

(14) Stukowski, A. Visualization and analysis of atomistic simulation data with OVITO–the Open Visualization Tool. Modelling and simulation in materials science and engineering, **2009**, 18(1), 015012.
